# Neurological manifestations of encephalitic alphaviruses, traumatic brain injuries, and organophosphorus nerve agent exposure

**DOI:** 10.3389/fnins.2024.1514940

**Published:** 2024-12-13

**Authors:** Morgen VanderGiessen, Caroline de Jager, Julia Leighton, Hehuang Xie, Michelle Theus, Erik Johnson, Kylene Kehn-Hall

**Affiliations:** ^1^Department of Biomedical Sciences and Pathobiology, Virginia-Maryland College of Veterinary Medicine, Virginia Polytechnic Institute and State University, Blacksburg, VA, United States; ^2^Center for Emerging, Zoonotic, and Arthropod-borne Pathogens, Virginia Polytechnic Institute and State University, Blacksburg, VA, United States; ^3^Translational Biology Medicine and Health Graduate Program, Virginia Tech, Blacksburg, VA, United States; ^4^Neuroscience Department, Medical Toxicology Division, U.S. Army Medical Research Institute of Chemical Defense, Aberdeen Proving Ground, MD, United States

**Keywords:** Venezuelan equine encephalitis virus, eastern equine encephalitis virus, western equine encephalitis virus, neuroinflammation, traumatic brain injury, organophosphorus nerve agent, neurological sequelae

## Abstract

Encephalitic alphaviruses (EEVs), Traumatic Brain Injuries (TBI), and organophosphorus nerve agents (NAs) are three diverse biological, physical, and chemical injuries that can lead to long-term neurological deficits in humans. EEVs include Venezuelan, eastern, and western equine encephalitis viruses. This review describes the current understanding of neurological pathology during these three conditions, provides a comparative review of case studies vs. animal models, and summarizes current therapeutics. While epidemiological data on clinical and pathological manifestations of these conditions are known in humans, much of our current mechanistic understanding relies upon animal models. Here we review the animal models findings for EEVs, TBIs, and NAs and compare these with what is known from human case studies. Additionally, research on NAs and EEVs is limited due to their classification as high-risk pathogens (BSL-3) and/or select agents; therefore, we leverage commonalities with TBI to develop a further understanding of the mechanisms of neurological damage. Furthermore, we discuss overlapping neurological damage mechanisms between TBI, NAs, and EEVs that highlight novel medical countermeasure opportunities. We describe current treatment methods for reducing neurological damage induced by individual conditions and general neuroprotective treatment options. Finally, we discuss perspectives on the future of neuroprotective drug development against long-term neurological sequelae of EEVs, TBIs, and NAs.

## 1 Introduction and global impact

### 1.1 EEVs

The equine encephalitis complex, including western, eastern, and Venezuelan equine encephalitis viruses (WEEV, EEEV, and VEEV), are New World alphaviruses in the family *Togaviridae* (Aguilar et al., 2011; Calisher, 1994). These viruses are collectively referred to as equine encephalitic viruses (EEVs) throughout this manuscript. These viruses are maintained in an enzootic cycle between mosquitoes and an animal host (e.g., rodents and/or birds) but can spill over into both humans and horses, causing illness (Ronca et al., 2016). In horses, mules, and donkey's, equine encephalitis can cause appetite loss, flu-like symptoms, and progress to muscle and nervous system degeneration (Guzmán-Terán et al., 2020). In humans, illness can include flu-like symptoms and can also progress to neurological deficits and seizures. These effects occur most commonly in children for whom EEVs are more likely to cause life-long behavioral deficits, seizures, and delirium (Falchek, 2012). Cases that progress to neurological deficits, including encephalitis require significant long-term care, which was estimated to cost $320,000 per individual in 1971, and $400,000 per year per individual in 1995 (Villari et al., 1995; Earnest et al., 1971). Based on the 2.54% inflation of the economy between 1995 and 2023 (Webster, 2024), this equates to ~$790,000 per year in 2023. Despite over 100 years of research on these viruses, there are no FDA-approved antiviral treatments or vaccines against EEV infection in humans. Ultimately, current treatment options are limited to supportive care. The lack of FDA approved therapeutics for EEVs is due to a variety of challenges, including the limited number of laboratories researching these viruses because of biosafety level limitations and the lack of adequate and standardized animal models.

### 1.2 TBI

Traumatic brain injury (TBI) is a leading cause of death and disability across the world, with an estimated 69 million people sustaining an injury each year (Dewan et al., 2018; Peterson et al., 2022). The CDC defines a TBI as “an injury that affects how the brain works” and may be caused by a “bump, blow, or jolt to the head, or a penetrating injury” (CDC, 2023a). Following the primary insult, “secondary injury” will occur in the minutes, hours, days, weeks, and years' post-injury. Secondary injury leads to chronic neurological deficits due to cell death, inflammation, and other consequences of primary injury. Additionally, TBI is a risk factor for several neurodegenerative disorders and neurological disorders and impairments (Dams-O'Connor et al., 2016; Delic et al., 2020; Rapoport, 2012; Howlett et al., 2022). Both primary and secondary injury lead to acute seizures in 1/5 individuals who receive a TBI and chronic post-traumatic epilepsy (PTE) occurs in ~1/50 cases (Fordington and Manford, 2020). Due to the heterogeneous nature of the TBI mechanism, pathophysiology, severity, and outcome, developing effective therapeutic strategies and treatments has yielded limited success, with no correct or universal FDA-approved treatments available (Kochanek et al., 2020; Nishimura et al., 2022). Several recent review articles and reports cover new technological advances in TBI research (Bowman et al., 2022; Bonanno et al., 2022; Lu et al., 2015; Ahmed, 2022). These focus on developments in imaging, biomarkers, and therapeutic approaches that offer insights into the pathophysiology and treatment of TBI. For instance, innovations in high-density neurophysiology monitoring systems, alongside advanced neuroimaging and bioinformatics, have enabled more precise tracking of brain injury progression and recovery. Technologies like high-resolution MRI, PET imaging, and blood-based biomarkers are helping researchers characterize injury severity and predict outcomes more accurately. Therefore, novel approaches to studying TBI and its related pathophysiology may enable therapeutic development.

### 1.3 NAs

Organophosphate compounds (OPs) are primarily divided into nerve agent OPs and pesticide OPs. Exposure to OPs causes the death of 300,000 people per year worldwide (Ahmad et al., 2024; Adeyinka et al., 2024), primarily from occupational exposure to pesticide OPs and exposures in underdeveloped countries. Challenges in identification of agent in a warfare setting make estimation of deadly nerve agent OP exposures difficult and likely underestimated (Costanzi et al., 2018; Gunnell et al., 2007) though there are recent high-profile examples of nerve agent OP use on civilians (Chai et al., 2017; Haslam et al., 2022; John et al., 2018; Morita et al., 1995). For this review we will focus on nerve agents (NAs) as these have significant implications as warfare agents (Mukherjee and Gupta, 2020). NAs irreversibly inhibit acetylcholinesterase activity, causing a buildup of acetylcholine in both the central and peripheral nervous systems that produces significant neurological changes (Costanzi et al., 2018). NAs are typically broken into classes, including the “German” G-series agents sarin (GB), soman (GD), tabun (GA), and the Russian “Venomous, Victory, or Viscous” V-series agents, which consists of VE, VG, VM, VR, and VX. Nerve agent mechanisms of action are consistent across the series, but the volatility and toxicity vary. V-series nerve agents, most notably VX, are less volatile at ambient temperature and generally regarded as more toxic than G-series agents, but this toxicity is typically associated with skin contact and persistent environmental hazard (Jang et al., 2015; Wiener and Hoffman, 2004; Rosenblatt et al., 1996). G-series agents are volatile at room temperature leading to more significant toxicity through inhalation or skin contact. An additional class, the Novichoks and A-series agents, will not be discussed in this review as their classification, chemical structures, and mechanisms are not well characterized by the literature. The little information that is present concerning this series is incomplete and from scrutinized sources, whereas G-series and V-series agents are relatively well characterized in literature. A-series and Novichok agents in particular display an extreme level of toxicity not observed in other nerve agents (Opravil et al., 2023; Noga and Jurowski, 2023).

Exposure to NAs can occur through inhalation, ingestion, or skin absorption (Wiercinski and Jackson, 2024). Small amounts of these chemicals can cause a variety of mild to moderate flu-like symptoms such as nausea, vomiting, confusion, headache, and weakness or other cholinergic symptoms such as watering eyes, drooling, blurred vision, increased heart rate, muscle spasms, and sweating (CDC, 2023b). The onset of symptoms is typically rapid at high doses, but long-term low dosage exposure can induce memory impairment, motor dysfunction, depression, and anxiety, with multiple lines of evidence from Tokyo Subway Attack victims, veterans, and exposed Iraqi civilians (Figueiredo et al., 2018). The mechanism by which long-term low-dose exposure of NA induces depression and behavioral changes is not well understood: however, neuronal damage and degeneration is commonly correlated with cognitive and motor dysfunction observed post-mortem (Figueiredo et al., 2018). Lower doses of NAs also can cause convulsion mediated at peripheral neuromuscular junctions, high levels of NA exposure almost invariably result in prolonged or repetitive central seizures (status epilepticus, SE) or death without immediate medical intervention (Hrvat and Kovarik, 2020). Unique to NAs, most long-term neurological changes are due to SE-induced neuronal damage, inflammation, and changes to neuronal signaling (Figueiredo et al., 2018) as NAs do not have directly toxic effects on neural cells (Aroniadou-Anderjaska et al., 2023).

## 2 Neuropathology in humans and laboratory animal models

### 2.1 EEVs

Human case studies of VEEV, EEEV, and WEEV are extensively under-documented due to the initial symptoms often presenting as febrile illness as well as non-specific documentation due to these viral illnesses being classified under a larger umbrella of neuroinvasive viruses, including flaviviruses, West Nile virus (WNV), and Japanese encephalitis virus (JEV) (Calisher, 1994; Ronca et al., 2016). The number of reported VEEV, WEEV, and EEEV cases trace back to the 1930s, the same time frame in which these viruses were isolated and mosquito vectors were determined to be the route of transmission (Weaver et al., 2004a; Zacks and Paessler, 2010). The combined impact of WEEV, VEEV, and EEEV includes >300,000 cases, >300 recorded deaths, and 3,000 survivors with long-lasting neurological disorders, including paralysis, seizures, migraines, and depression (Crosby and Crespo, 2024) (Table 1). As previously mentioned, this is likely an underestimate due to many viral infections causing vague symptoms in humans, and therefore these infections may go undiagnosed and untested. A recent study identified elevated glial fibrillary acidic protein (GFAP) in patient serum from acute VEEV and Madariaga virus (MADV) infections; however this biomarker was not specific to alphavirus infection as bacterial cases with encephalitis also displayed elevated GFAP (Bartlett et al., 2024). EEV pathology in humans has been divided into three distinct phases consisting of a lymphotropic phase (early/short term), a neuroinvasive phase, and a neurodegenerative phase (late/long-term) [reviewed in Steele and Twenhafel (2010); Kehn-Hall and Bradfute (2022)]. Neurological complications can be attributed to both the neuroinvasion and replication of EEVs in the brain, as well as from the inflammatory response. Despite the large range of neurological deficits induced by these viruses, there is little known about the mechanism of neuroinvasion in humans. Similarly, it is debated whether the inflammatory process hallmarked by the increase of neutrophils in the central nervous system (CNS) is harmful or helpful (Peiseler and Kubes, 2019; Drescher and Bai, 2013). Therefore, we rely upon animal models of infection to study pathologic, behavioral, and molecular processes altered by infection and ultimately identifying therapeutics. Animal models of EEVs have previously been reviewed (Ronca et al., 2016; Steele and Twenhafel, 2010; Kehn-Hall and Bradfute, 2022); however, here, we seek to highlight the consequences of neuroinvasion and long-term neurological symptoms of EEVs in order to correlate them across different neurological diseases.

**Table 1:** Impacts of EEVs, TBI, and NA on humans.

	**EEV**	**TBI**	**NA**
Case Fatality	30%−75% (EEEV), 1% (VEEV), and 3%−7% (WEEV)	2%−4%	0%−50% >300,000 OP pesticide exposures per year worldwide
Case incidence	>300,000 (VEEV), >400 (EEEV), >600 (WEEV) total cases, but likely more due to mild illness and minimal testing	69 million per year worldwide	>0 per year 3 million OP pesticide exposures per year worldwide
% Progress to neurological sequelae	50%–90%, (EEEV), 4–14% (VEEV), and 15%–30% (WEEV)	15%−30% and 10%−50% of severe TBI are at risk of developing epilepsy Children are at a higher risk for developing PTE than adults	10%−40%
References	Ronca et al., 2016; Guzmán-Terán et al., 2020; Zacks and Paessler, 2010; Carrera et al., 2018	Dewan et al., 2018; Peterson et al., 2022; Frey, 2003; Hahn et al., 1988; Amare et al., 2021; Demlie et al., 2023	Wiercinski and Jackson, 2024; Aroniadou-Anderjaska et al., 2016

Case fatality, incidence and progression to neurological disease is highly variable across different neurological diseases despite similar symptom manifestations.

Animal models used for EEV research include non-human primates, rats, gerbils, mice, guinea pigs, rabbits, and hamsters, which have been extensively reviewed by others (Steele and Twenhafel, 2010; Kehn-Hall and Bradfute, 2022; Dremov and Solianik, 1977). Of these models, Guinea pigs, hamsters, and rabbits have high fatality rates, including severe lymphoid necrosis prior to invasion into the CNS; therefore, they are less ideal models for studying neurological sequelae. In this review, we focus on rodents (i.e. mice, rats, and some hamster models) and non-human primates (NHPs) as animal models of EEV infection, where neurological infiltration has been well established. It's also important to note that studies in animal models have primarily been reliant upon the utilization of a variety of different strains of VEEV, EEEV, and WEEV, including naturally evolving strains (VEEV Subtypes I-VI, VEEV Trinidad Donkey (TrD), EEEV Georgia Fatal, EEEV FL93-939, EEEV North American, WEEV MacMillan (MCM), WEEV IMP 181) as well as some attenuated viral isolates previously reviewed (e.g. VEEV TC-83) (Steele and Twenhafel, 2010; Sharma and Knollmann-Ritschel, 2019). Route of infection is also important to consider as there are multiple infection methods used including intranasal, aerosolization, and subcutaneous infection; however, there are different degrees of morbidity and mortality across the different infection routes. While EEVs are naturally transmitted via mosquito bite, there is a large interest in understanding pathogenesis that results from inhalational exposure. EEVs are readily aerosolized, VEEV was developed as a biological weapon, and a large number of laboratory acquired infections have occurred via VEEV aerosolization (Rusnak et al., 2018; Weaver et al., 2004b).

#### 2.1.1 VEEV

VEEV infections in humans result in a relatively low mortality rate (< 1%); however, the progression to neurological deficits is significant with 4%−14% of cases progressing to neurological signs with a higher incidence in children, elderly, and immune compromised people (Lundberg et al., 2017). The two largest outbreaks of VEEV occurred through mosquito transmission in Texas in 1971 and Columbia in 1995 (Aguilar et al., 2011). Collectively, there were >300 deaths reported and 3,000 cases with long-lasting neurological disorders including paralysis, seizures, migraines, and depression (Crosby and Crespo, 2024). Additional symptoms included reduced sensory perception of taste, hearing, and smell and changes in emotional stability and mental fitness (Carrera J. P. et al., 2013; Bowen et al., 1976). These studies highlight significant infection rates and neurological sequelae resulting from VEEV outbreaks.

VEEV infection in NHPs results in clinical symptoms such as weight loss, lethargy, hunching, hyperactivity aggression, photophobia, and full body and partial tremors, which closely resembles infection in humans (Table 2) (Burke et al., 2019). Aerosolized VEEV exposure typically results in prolonged febrile state for 1–8 days post-infection (DPI) with immediate invasion into the brain, whereas subcutaneous administration typically presents as febrile illness for 1–6 DPI with signs of virus in the brain and neurological symptoms such as depression 2–3 DPI (Reed et al., 2004; Weaver et al., 2012; Gleiser et al., 1961; Ludwig et al., 2001). The intranasal route of exposure led to the most severe illness in NHPs with invasion, replication, and lesions due to viral infection as early as 48 h post infection (hpi). Further investigation of infected NHPs identified neuronal necrosis, lesions in the thalamus and olfactory cortex, and perivascular cuffing and gliosis with severe inflammation in the hippocampus and cortex (Reed et al., 2004; Gleiser et al., 1961; Danes et al., 1973; Victor et al., 1956; Smith et al., 2020). In Rhesus Monkeys, vertical transmission via fetal contraction of VEEV was confirmed via intranasal infection of the mother. The fetuses showed viral replication in both the brain and peripheral organs, which led to vision impairment and abnormal brain growth, including microcephaly, hydrocephalus, and porencephaly in 67% of cases (London et al., 1977). Evaluation of VEEV in NHP has several advantages as they display highly similar patterns of febrile illness, lethargy, and depression to human case studies; however, murine models of infection include several cost-effective and higher throughputs benefits not represented in this model.

**Table 2:** Comparison of human EEV cases and laboratory animal models of EEV infection.

**Species/strain**	**Virus and viral strain (if known)**	**Acute symptoms**	**Neurological manifestation**	**Pathological changes**	**References**
**Humans**					
N/A	VEEV	Flu-like symptoms, such as fever, chills, malaise, severe headache, myalgia in the legs and lower back, tachycardia, and in some cases, nausea, vomiting, and diarrhea, death or moribund	Convulsions, somnolence, confusion, photophobia, coma, intellectual disability, and emotional instability/behavioral changes, seizures, drowsiness, disorientation, depression	Cerebrovascular congestion, encephalitis, edema, inflammatory cell infiltrates, intracerebral hemorrhage, vasculitis, meningitis, cerebritis	Aguilar et al., 2011; Ronca et al., 2016; Guzmán-Terán et al., 2020; Casals et al., 1943; Lennette and Koprowski, 1943; de la Monte et al., 1985
N/A	EEEV	Fever, confusion, headache, shock, Neck pain, vomiting, chills, death or moribund	Stupor, left-sided weakness, thalamic enhancement, seizures, hemiparesis and psychomotor retardation, dysarthria Convulsions, seizures, paralysis, intellectual disability, vegetative state, and behavioral changes	Diffuse edema, herniation, hemorrhage, inflammatory infiltrates, vasculitis, necrosis, microglia activation, necrosis, thrombosis, hypoxic-ischemic changes	Ronca et al., 2016; Lindsey et al., 2020; Langsjoen et al., 2023; Carrera J. P. et al., 2013; Farber et al., 1940
N/A	WEEV	Fever, malaise, headache, nausea, vomiting, weakness, death or moribund	Seizure, neck stiffness, photophobia, Parkinson's-like syndrome	Intracranial hypertension, temporal ventricle dilation, intracranial hypertension, encephalitis	Delfraro et al., 2011
**Non-human primates**					
Macaque *(Macaca fascicularis)*	VEEV (TrD)	Fever, lethargy, death or moribund	N/A	Lymphopenia, lesions in olfactory cortex and thalamus	Steele and Twenhafel, 2010; Rusnak et al., 2018, 2019; Steele et al., 1998; Koterski et al., 2007
Macaque *(Macaca fascicularis)*	VEEV (INH-9813)	Weight loss	N/A	Neuronal necrosis, gliosis in the cerebellum, inflammation in the meninges, perivascular cuffing	Smith et al., 2020; Ma et al., 2022
Macaque *(Macaca fascicularis)*	EEEV (V105)	Nausea, vomiting, fever, general malaise, and/or headache (2–3 DPI), death or moribund	Seizures	Parenchyma lesions in the basal ganglia, thalamus, and cerebral cortex, mesencephalon, medulla oblongata, mild neuronal degradation, neuronal necrosis, neuropil vacuolation, gliosis, satellitosis, microhemorrhage, perivascular cuffing	Williams et al., 2022; Albe et al., 2021
Macaque *(Macaca fascicularis)*	EEEV (FL93–939)	Dehydration, lethargy, hyperthermia, increased salivation, ataxia, fever, death or moribund	Limb weakness and imbalance, tremors, immobility, abnormal vocalizations	Neutropenia, lymphopenia, leukocytosis, lesions in the cerebrum, cerebellum, and brainstem, inflammation in the meninges	Smith et al., 2020
Marmoset *(Callithrix jacchus)*	EEEV (FL93–939)	Weight loss, fever, anorexia, depression, fever	Imbalance, tremors, hypothermia	Meningoencephalitis, retinitis	Porter et al., 2017
Macaque *(Macaca fascicularis)*	WEEV (Fleming)	Fever	Tremors	Lymphopenia, leukocytosis, monocyte infiltration, encephalitis, meningoencephalitis, perivascular cuffing, hydrocephalus, perivascular cuffing, lymphoid hyperplasia	Smith et al., 2020; Dupuy and Reed, 2012
Macaque *(Macaca fascicularis)*	WEEV (CBA-87)	Fever	Tremors, convulsing, unresponsive, prostration, behavioral changes, reduced appetite, reduced activity	Not described	Burke et al., 2019; Reed et al., 2005
**Mice (*Mus musculus*)**					
C57BL/6	VEEV (TC-83)	Piloerection, hunching, weight loss	Reduced motor function	Inflammation, mononuclear cell infiltrates, meningitis, microglia activation, perivascular cuffing	Taylor et al., 2017
C3H/HeN	VEEV (TC-83)	Weight loss, lethargy, weakness, hunching, hypersensitivity, death or moribund	Circling, altered gait, hyperactivity, seizures, ataxia, piloerection	Necrosis in the olfactory epithelium, Pyknosis, karyorrhexis, reduced mature olfactory receptor neurons, encephalitis, lymphoid depletion, meningitis, perivascular cuffing	Rusnak et al., 2019; Steele et al., 1998; Taylor and Paessler, 2013; Williams et al., 2023, 2022; Taylor et al., 2012, 2017
CD-1	VEEV (V3000)	Hunching, ruffled fur, lethargy, shivering, death or moribund	Circling, hind limb paralysis	Perivascular cuffing, vessel thickening and endothelial cuffing (inflammation), neutrophil infiltration	Sharma et al., 2008; Aronson et al., 2000
BALB/c	VEEV (TrD)	Lethargy, decreased grooming and ruffled fur, hunched posture, death or moribund	Hind-limb paralysis	Spongiosis, neutrophil cellularity, neuronal death, perivascular cuffing, meningitis	Ludwig et al., 2001; Cain et al., 2023; Steele et al., 1998; Steele and Twenhafel, 2010; Rusnak et al., 2018; Cain et al., 2017; Jackson et al., 1991
CD-1	VEEV (ZPC738)	Ruffled fur, lethargy, weight loss, death or moribund	Altered mobility, paralysis, seizure, ataxia	ND	Gardner et al., 2008
C57BL/6	EEEV FL91–4679	Fever, lethargy, hunched, weight loss, ruffled fur. death or moribund	Prostration, tremors,	Neuronal necrosis, perivascular cuffing, inclusion bodies, and neuronal necrosis, Lesions and injury of basal ganglia, thalamus, and cortex, cerebral injury, death	Vogel et al., 2005; Schoepp et al., 2002
BALB/c	EEEV (FL93–939)	Hunching, weight loss, ruffled fur, death or moribund	Paralysis, non- responsiveness	Meningoencephalitis, neutrophil vacuolation, and gliosis	Honnold et al., 2015a,b
CD-1	EEEV (FL93–939)	Ruffled fur, lethargy, weight loss, death or moribund	Altered mobility, paralysis, seizure, ataxia, piloerection	Neuron, dendrites, and macrophage damage	Gardner et al., 2008
BALB/c	WEEV (Flemming)	Hunched posture, piloerection, reduced mobility, lateral and ventral recumbency, death or moribund	Seizures, fixed gaze, reduction in motor control, reduced response to stimuli	Reduced neurons, shrunken neurons, perivascular cuffing, vacuolation of hippocampus, neuronal death, Pyknosis, karyorrhexis	Phelps et al., 2017, 2021
C57BL/6	WEEV (MacMillan strain)	ND	ND	A-synuclein protein aggregation in the cortex, hippocampus, midbrain, microgliosis, astrogliosis, dopaminergic neuron loss	Bantle et al., 2019, 2021

ND, not described.

**Figure 1:**
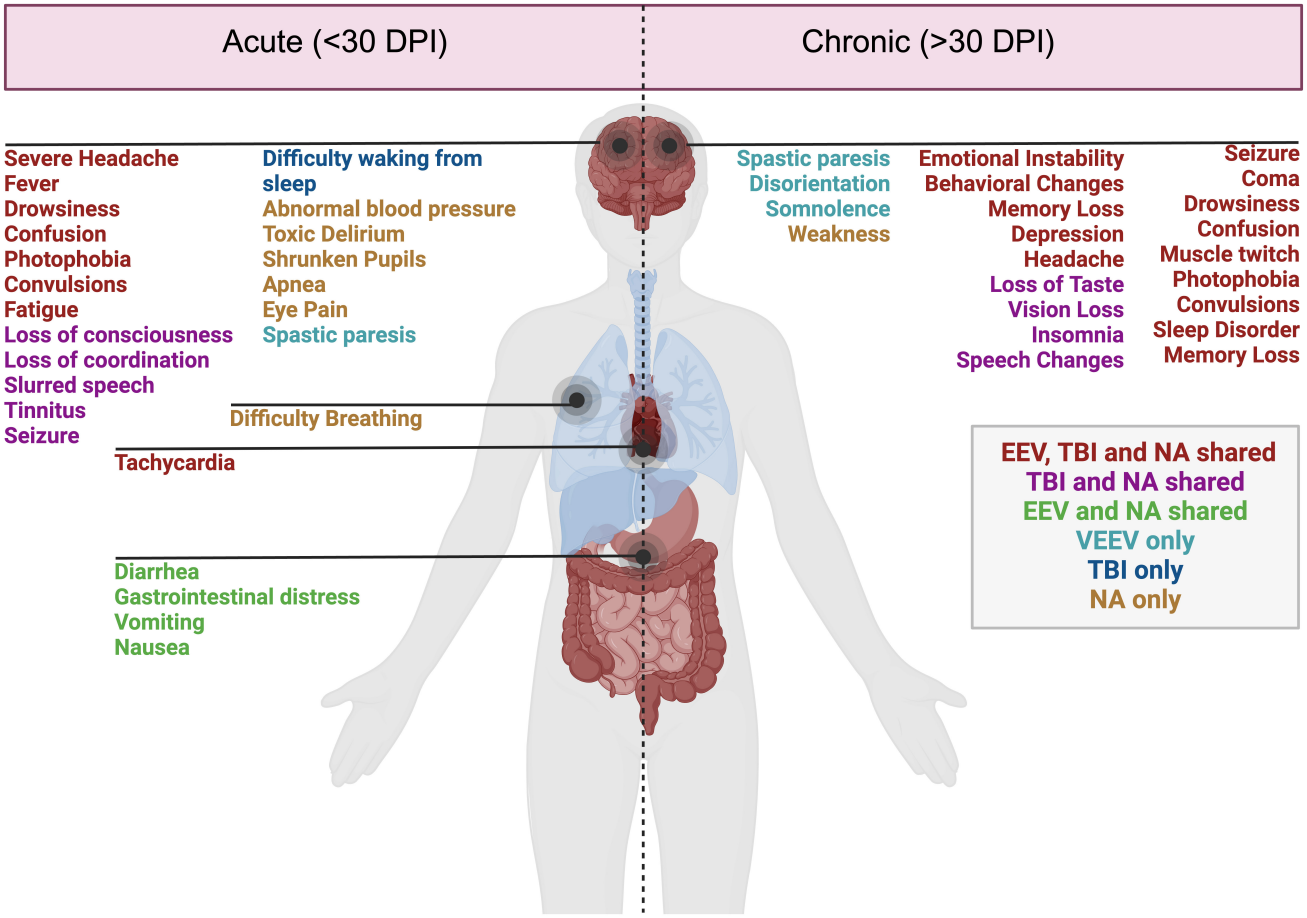
Acute and chronic clinical comparisons between EEVs, TBI, and NA. Summary of characterized acute (< 30 days post-injury or exposure) and chronic manifestations (>30 days post-exposure) of disease in humans. Overlapping clinical symptoms and sequelae are color-coded. EEV, TBI, and NA (red), TBI and NA shared (magenta), VEEV and NA shared (green), VEEV only (teal), TBI only (blue), and NA (gold). DPI, days post injury/infection. Created in BioRender. Kehn-hall (2024) BioRender.com/r18b546.

Infection in mice via footpad or subcutaneous injection leads to viral replication in the lymphatic system as early as 4 hpi, viremia is detected at 12 hpi, and virus in the brain between 24–72 hpi whereas aerosolization or intranasal infection leads to infection in the brain 16–48 h post-infection (Davis et al., 1994; Rusnak et al., 2019). Multiple strains of mouse models (C57Bl/6, CD-1, BALB/c, C3H/HeN) have been well established with intranasally VEEV-infected mice showing signs of weight loss, tremors, paralysis, and dehydration (Table 2) (Gardner et al., 2008; Berge et al., 1961; Hart et al., 1997, 2000; Vogel et al., 2005; Honnold et al., 2015a,b). In most models of VEEV infection, there is direct entrance into the brain via the olfactory bulb via aerosolization and intranasal routes, which has aided in further understanding the neurological features of infection (Cain et al., 2023; Phillips et al., 2016; Salimi et al., 2020). VEEV can also enter the brain via transcytosis through brain epithelium, independent of blood-brain barrier (BBB) breakdown, which appears later in the course of infection (Salimi et al., 2020). Caveolin-1 was shown to be important for viral neuroinvasion (Salimi et al., 2020). VEEV infection typically results in 100% mortality in mice when they are infected with fully virulent BSL-3 strains of VEEV. However, VEEV TC83 is a live attenuated vaccine strain that is used to study VEEV pathogenesis at BSL-2. VEEV TC83 infection results in different levels of mortality dependent on the strain of mouse utilized. C3H/HeN mice are highly susceptible to VEEV TC83, resulting in 100% mortality when intranasally infected with 10^9.1^ and 10^7.1^ cell culture infection dose 50 (CCID50) (Julander et al., 2008). It's been proposed that higher mortality rates in VEEV-infected C3H/HeN mice compared to C57BL/6 and BALB/c mice is due to a reduced immune response partially dependent on reduced IgA in C3H/HeN mucosa (Hart et al., 1997; Steele et al., 1998; Charles et al., 1997). The majority of C57BL/6 mice infected intranasally with VEEV TC-83 survive infection and serve as an important model to study neurological sequelae following VEEV infection (Ronca et al., 2017; Cain et al., 2017). These mice display biphasic disease initially causing symptoms such as ruffled fur, weight loss, lethargy, and shivering (Figure 1). Following neuroinvasion, circling, hind limb weakness or paralysis, convulsions, and head-tilt are observed. Post-mortem analysis of VEEV V3000 (molecular clone of fully virulent VEEV TrD) infected mice indicated perivascular cuffing, inclusion bodies, neuronal necrosis, and overt encephalitis widespread across the brain (Sharma and Knollmann-Ritschel, 2019; Cain et al., 2023). It has been confirmed that in mice, VEEV primarily infects neurons, but also infects microglia, macrophages, and oligodendrocytes as well as inducing significant inflammation around the BBB (Sharma and Knollmann-Ritschel, 2019).

There has been a significant effort to determine transcriptomic changes associated with VEEV infection, although much has been done in cell culture with increasing evaluation in murine models. In VEEV-infected mice, neuronal damage has been correlated with upregulation of apoptotic, antiviral, T-cell response, and pro-inflammatory genes including CXCL10, IL1-beta, Interferon gamma (IFN-y), CCL5 (RANTES), CCL3 (MIP-1a) and TNF-alpha (Weaver et al., 2004a; Sharma and Knollmann-Ritschel, 2019; Sharma et al., 2008; Taylor and Paessler, 2013). Symptomatic mice appear to have more significant upregulation of inflammatory genes, as well as signs of increased astrocyte activation within specific areas of the brain, such as the thalamus and hippocampus, even after 6 months post-infection (Ronca et al., 2016). A more expansive study investigated individual areas of the brain on days 1–7 post-infection and identified upregulation of numerous cell death pathways (phagocytic pathways, natural killer, pyroptosis, necroptosis) and pro-inflammatory response (Williams et al., 2023). Collectively, these studies have established that there is much similarity between humans, NHPs, and mice with CNS invasion leading to markers of encephalitis such as perivascular cuffing, neuronal necrosis, as well as other markers of damage including infiltration of leukocytes, brain lesions, and gliosis. Evaluation of proteomic and metabolomic changes is currently limited to cell culture-based studies of VEEV and is thus not included in this review.

#### 2.1.2 EEEV

EEEV infections result in 30%−75% mortality and 50%−90% of surviving individuals progress to long-term neurological symptoms (Lindsey et al., 2020; Ciota, 2022; Langsjoen et al., 2023). Collectively, chronic symptoms are poorly characterized in EEV infected animals due to the high mortality rate. Small periodic cases of EEEV and the emergence of four diverse EEEV lineages from 1960–2010 have yielded inconsistent reporting of morbidity, mortality, and neurological sequelae (Arrigo et al., 2010). There were ~178 documented cases of EEEV infection from 2009–2020 with the majority of these cases in Panama and the eastern United States, with the highest number of cases in Georgia, New Jersey, New York, North Carolina, and Michigan (Carrera J. P. et al., 2013; Virmani et al., 2010; Hill et al., 2023; Vilcarromero et al., 2010). Surveillance efforts identified 100 potential positive cases and seven confirmed cases of EEEV in Panama in 2010, where many survivors experienced chronic seizures which were traced to virally induced abnormalities within the temporal lobe of the brain, responsible for memory storage, formation, sensory processing, and emotions (Carrera J. P. et al., 2013). Sequelae observed in human cases include behavioral, memory, and emotional changes which are potentially attributed to changes such as neuronal loss, vasculitis, thrombosis, gliosis in motor neurons, and abnormalities in the temporal lobe (Table 2) (Ronca et al., 2016; Reddy et al., 2008).

In NHP models of EEEV infection, high fatality rates have been recorded in correlation with the high fatality rates seen in humans (Williams et al., 2022). Studies in NHPs have identified EEEV replication in the CNS and severe neurological disease (Albe et al., 2021; Ma et al., 2022). NHPs infected with EEEV display neuronal dysfunction in the olfactory bulb, olfactory tract, and spinal cord which is associated with EEEV pathology rather than neuronal death, which is also typically seen in VEEV. An overall review of NHP EEEV infections has identified pathological evidence of vasculitis, perivascular cuffing, edema, hemorrhage, and widespread necrosis widespread across the brain (Steele and Twenhafel, 2010; Reed et al., 2004; Arrigo et al., 2010). Another unique characteristic of EEEV in Cynomolgus macaques is the neuroinvasion and pathology in the brain is equally as severe between both intranasal and subcutaneous injection, potentially due to EEEV entering the brain via the bloodstream rather than olfactory nerve routes (Smith et al., 2020).

Mouse models of EEEV are also relatively understudied. EEEV is highly fatal in mice, typically 100% in all routes of infection with accompanying invasion into the brain even with subcutaneous methods as early as 24 h post-infection (Vogel et al., 2005). EEEV infected mice show preferential infection of osteoblasts, skeletal muscle, and fibroblasts, with less efficient replication in macrophages and dendritic cells (Steele and Twenhafel, 2010; Gardner et al., 2008; Vogel et al., 2005). Cytokine analysis indicated CCL5 (RANTES), CXCL9 (MIG), CCL4 (MIP-1B), and IFN-y peak between day 1 and 2 post infection dependent on the route of infection (Honnold et al., 2015a). In CD-1 mice infected with 10^3^ plaque forming units of either VEEV or EEEV, both viruses showed similar trends with decreased weight, piloerection, paralysis, seizure, and 100% mortality by day 7 (Gardner et al., 2008). Disease symptoms of VEEV onset earlier with mice surviving longer with clinical signs of CNS infection; whereas EEEV-infected mice showed later onset of symptoms and mortality within 12–24 h post disease onset (Gardner et al., 2008). Potentially, this is driven by cellular tropism differences between VEEV and EEEV, as EEEV replicates poorly in lymphoid tissues while VEEV flourishes in macrophages and dendritic cells in these tissues (Gardner et al., 2008). The overarching similarity between VEEV and EEEV infections in mice are encephalitis; however, in VEEV-infected mice, neurons in the hippocampus and cerebellum show signs of morphological changes correlated with apoptosis, whereas in EEEV seem to infiltrate the thalamus, pons, and putamen with widespread neuronal necrosis (Vogel et al., 2005). There are a few consistent differences noted with VEEV and EEEV infection in mice which has not been fully elucidated in NHP and humans where paralysis is more common in VEEV and have a rarer occurrence of seizures, while with EEEV there are few cases of paralysis and frequent report of seizures (Gardner et al., 2008). Collectively, EEEV pathogenesis and neuroinvasion appear to be more rapid, although EEEV infected mice display widespread neurological damage and proinflammatory activation similar to VEEV with slight alterations dependent on the route of exposure.

#### 2.1.3 WEEV

Several hundred cases of WEEV infection have been well documented, with neurological sequelae lingering for several years, if not lifelong, post-infection. In children and some adults, mild sequelae include loss of taste, changes in speech, decreased fine motor skills, changes in gait, and hearing deficits (Table 2) (Mulder et al., 1951). Severe sequelae in both adults and children include personality and behavioral changes, intellectual disability, seizure, limb weakness, mood swings, depression, anxiety and paranoia (Palmer and Finley, 1956; Fulton and Burton, 1953; Deaton et al., 1986). Neurological manifestation of WEEV infections are the most significantly documented partially attributed to its lower mortality (< 7%) and high incidence of neurological symptoms (15%−30%) (Luethy, 2023; Simon et al., 2023). Documentation of WEEV in an aerosol laboratory-acquired infection lead to the death of 2/5 infected individuals following symptoms such as headache, fever, tachycardia, and increased heart rate (Hanson et al., 1967). A recent outbreak of WEEV occurred in early 2024, where >100 equine cases, most of them fatal, and >100 human cases were documented with 10 fatalities (Campos et al., 2024). Campos et al. (2024) identified a novel WEEV lineage likely due to unreported cases circulating in South America, but it does not currently appear to be a recombination with other EEVs. In this outbreak, most cases were mild or asymptomatic, with signs or meningitis and encephalitis in fatal cases (Campos et al., 2024). Interestingly, some patients with WEEV have been described to have other neurological symptoms that mirror those diagnosed with diseases such as Parkinson's and Schizophrenia (Bantle et al., 2019; Levine and Griffin, 1992; Herzon et al., 1957). One recent case occurred in November 2023 of an equine agricultural working in Argentina, where the patient presented with headache, fever, disorientation, confusion, and tiredness, leading to intensive care treatment for 12 days and eventual discharge nearly 30 days after initial symptoms (World Health Organization, 2023). Given both a recent case and severity of disease across many documented cases, it highlights the need for adequate surveillance, diagnosis, and animal models for therapeutic evaluation against WEEV.

WEEV infections in animal models have substantial research in murine, hamster, and NHP models. Studies of WEEV in NHPs have been minimal since the 1930s. Fever and increased heart rate, as well as clinical signs suggesting encephalitis, were observed in NHPs exposed to WEEV via aerosol (Smith et al., 2020; Reed et al., 2005). Some conflicting reports indicate minimal clinical signs and no recoverable virus from WEEV infected NHPs, but potentially this is due to quicker clearance compared to the other EEVs (Burke et al., 2022). Pathology from infected NHPs has indicated viral infection broadly across the brain with widespread infiltration in the gray matter of the brain in areas of the striatum and cerebrum and infection of the neurons in the hippocampus (Reed et al., 2005). Some models of WEEV and VEEV in NHPs suggest peak neutrophil and monocyte infiltration is slightly later around 7–9 DPI. Despite several studies that have investigated WEEV neuropathology, there is still much left unknown about the course of disease in NHPs, highlighting a significant gap in the literature.

Golden hamsters infected with WEEV resulted in 100% mortality with symptoms such as severe respiratory challenges, blurred cornea, eye discharge, and seizure with confirmation of the virus in the brain within 24 h of infection (Zlotnik et al., 1972). Older studies have revealed neuronal necrosis, edema, glial nodes, perivascular cuffs, and astrocytosis in mice 4–8 weeks old with 100% mortality in 2-day-old mice due to severe inflammation and necrosis of muscle, cartilage and bone marrows prior to neuroinvasion (Aguilar et al., 2011; Phillips et al., 2016; Gardner et al., 2022; Phelps et al., 2017). Suppression of WEEV replication in CD-1 mice using immunotherapy enabled the survival of mice and analysis of neurological sequelae (Bantle et al., 2019). These mice displayed Parkinson's-like symptoms and pathology seen in humans, including a loss of dopaminergic neurons, increased protein aggregation, and persistent neuroinflammatory responses (Bantle et al., 2019). In this model, WEEV replication was confirmed in the olfactory bulb, the cortex, hippocampus, and basal midbrain by day 4 post-infection with prolonged inflammatory response and glia and astrocyte activation 2 months post-infection. A variety of new literature has come out regarding WEEV pathology, especially surrounding its similarities to Parkinson's disease and the chronic consequences of neuroinvasion. Microgliosis, astrogliosis, dopaminergic neuron loss, and a-synuclein protein aggregation are observed across the brain with particular accumulation in the hippocampus and cortex (Bantle et al., 2021).

Despite vast advances in our understanding of disease induced by EEV family members, there have been no studies that have been able to correlate transcriptomic and histopathological features with specific changes in physical, emotional, and behavioral changes seen in humans. Collectively, given the vast similarities indicated in the acute phase of infection, it's likely that therapies could enhance protection against EEV fatality or neurological sequelae.

### 2.2 TBI

The National Institute of Neurological Disorders and Stroke classifies TBI as an external mechanical force that causes damage to the brain, potentially leading to temporary or permanent disability (NINDS, 2024). The most recent data suggests that over 200,000 TBI related hospitalizations occurred in 2020 and almost 70,000 TBI related deaths occurred in 2021 (CDC, 2024). TBI injury severity varies greatly both across human patients requiring varied animal models to replicate the disease processes and outcomes seen in people. TBI severity can be stratified by the Glasgow Coma Scale (GCS) into mild, moderate, and severe injuries (Vella et al., 2017; Jain and Iverson, 2023). Much of the human research conducted focuses on sports related injuries or combat related injuries (Fehily and Fitzgerald, 2017; Kim et al., 2023; Elder and Cristian, 2009). Even so, the majority of TBIs are caused by falls and frequently impact elderly individuals and young children (Faul et al., 2010). Further, males are over three times more likely to sustain a TBI than females, likely due to more prevalent high-risk behaviors and higher-risk jobs as this sex difference is only present in adults, not children (Alexis et al., 2022). The TBI injury cascade can be generally split into two categories: primary and secondary injury (Davis, 2000). The primary injury reflects the initial mechanical insult, such as axonal shearing, contusion, laceration, and skull fracture. The secondary injury includes the physiological aftermath that causes continued cell damage and death in the hours, days, and years following the initial impact. This includes BBB disruption, hypotension, hyperglycemia, hypoglycemia, changes in intracranial pressure, cerebral edema, peripheral immune cell infiltration, gliosis, and release of excitatory neurotransmitters. These mechanisms lead to the symptoms and outcomes seen in patients and replicated in animal models (Table 3). To replicate human TBI cases, animal models of TBI are adapted to study various injury types and severities (Xiong et al., 2013).

**Table 3:** Comparison of human TBI cases and laboratory animal models of TBI.

**Species**	**Injury model**	**Injury type**	**Acute symptoms**	**Neurological manifestations**	**Pathological changes**	**References**
**Humans**						
N/A	Repeated mild	Diffuse TBI	Post traumatic amnesia, loss of mental alertness, sometimes a loss of consciousness, anterograde amnesia, confusion, speech and gait abnormalities, personality changes	Mild cognitive impairment, earlier onset of Alzheimer's disease, more likely to suffer additional concussive injuries, eventual development of chronic traumatic encephalopathy (CTE)	Decreased cerebral blood flow, glucose metabolic dysfunction, intracranial pressure, neurofibrillary tangles, neuropil threads, gliosis, amyloid plaques, ventricular dilation, tau-immunoreactive astrocytes	Broussard et al., 2018; Guskiewicz et al., 2005; Collins et al., 2002; Guskiewicz et al., 2003; Omalu et al., 2006, 2005; McKee et al., 2009
N/A	Mild	Diffuse TBI	Loss of consciousness < 30 min, dizziness, confused, seeing stars, no memory of the injury, depression (can be chronic as well), lacking energy, fainting, increased anger	Cognitive impairment, psychiatric illness including schizophrenia, depression, hallucinations, anxiety, substance abuse, somatoform disorder, adjustment reaction, affective disorder, general psychiatric diagnosis, difficulty learning, difficulty problem solving, trouble concentrating, post traumatic epilepsy	BBB disruption, axonal injury, amyloid precursor protein accumulation, glial proliferation indicated by magnetic resonance spectroscopy (MRS), metabolic dysfunction, inflammation, macrophages and lymphocytes in the white matter, hemorrhagic lesion, microbleeds in the cortex, reduced magnetization transfer ratio (MTR) in the corpus callosum	McGowan et al., 2000; Garnett et al., 2000a,b; Brooks et al., 2000; Fann et al., 2004; Tomkins et al., 2011
N/A	Moderate	Diffuse or focal TBI	Loss of consciousness 30 min-6 h, dizziness, confused, seeing stars, no memory of the injury, persistent or worsening headache, vomiting, nausea, seizures, dilations of pupils, fluid draining from the nose or ears, inability to waken from sleep	Cognitive impairment, psychiatric illness including schizophrenia, depression, hallucinations, anxiety, substance abuse, somatoform disorder, adjustment reaction, affective disorder general psychiatric diagnosis	Hippocampal volume loss, increased serum cytokine levels, cognitive impairment, elevated cerebral spinal fluid (CSF), GFAP, MBP, and neurofilament light (NFL) deposition	Fann et al., 2004; Green et al., 2023; Milleville et al., 2021; Olczak et al., 2018
N/A	Severe	Diffuse or focal TBI	Loss of consciousness >6 h, dizziness, confused, seeing stars, vomiting, nausea, seizures, dilations of pupils, fluid draining from the nose or ears, inability to waken from sleep	Mood disorder, motor aphasia, inability to waken from sleep, negative corneal response, no memory of the injury, persistent or worsening headache, psychiatric illness including schizophrenia, depression, hallucinations, anxiety, substance abuse, somatoform disorder, adjustment reaction, affective disorder, general psychiatric diagnosis, loss of balance, slurred speech, weakness of limbs	Subarachnoid hemorrhage, subdural hematoma, extradural hematoma, intraventricular hemorrhage, effacement of ventricles, cerebral edema, bradycardia, elevated blood pressure and intracranial pressure, midline shift, cerebral contusion, skull fracture, cerebellar damage, BBB disruption	Fann et al., 2004; Nelson et al., 2016; Alexis et al., 2018; Saw et al., 2014; Ho et al., 2014
N/A	Blast injury	Diffuse TBI	Headache, insomnia, anxiety, memory impairment, depression, seizure disorder, chronic pain, altered vision or hearing, fatigue, sensitivity to light or sound, spatial memory impairment	Jumbled speech, abnormally slow hand movements	Sometimes no brain abnormalities are found post mortum, however, the following have been found in some cases: astroglial scarring and gliosis, CD68 staining of macrophages or microglia, abnormally phosphorylated tau, axonal spheroids, amyloid precursor protein buildup	Shively et al., 2016
N/A	Penetrating ballistic-like brain injury	Focal TBI	Loss of consciousness, headache, seizures, altered vision or hearing, pupil dilation, fluids draining from nose or ears, nausea and vomiting, confusion, dizziness, mood swings, fatigue, sensitivity to light or sound	Loss of balance, slurred speech, weakness of limbs, cognitive impairment	Hematoma, skull fracture, subarachnoid hemorrhage, ventricle narrowing, midline shift, cerebral edema, elevated intracranial pressure, contusions, CSF leakage, metabolic impairment	Wyck et al., 2015
**Non-human primates**						
Macaque *(Macaca mulatta)* Monkey (*Saimiri spp.)*	Controlled cortical impact (CCI)	Focal TBI	ND	Reduced mobility and dexterity	Increase in intracranial pressure. Loss of white matter, loss of gray matter	Barbay et al., 2021; King et al., 2010
Monkey/ Macaque (*Macaca mulatta*)	Diffuse axonal injury	Diffuse TBI	coma	Neurological impairment	Diffuse axonal injury in corpus callosum and brainstem	Gennarelli et al., 1987
Macaque (*Macaca fascicularis*)	Blast Injury	Blast	ND	Motor impairment, short term memory loss, loss of motor dexterity	Astrocyte hypertrophy, cerebral edma, apoptosis of astrocytes and oligodendrycytes, structural changes in purkinje (cerebellum) and pyramidal neurons (hippocampus), decreased white matter	Lu et al., 2012
Monkey/ macaque	Weight drop	ND				
Monkey/ macaque	Fluid percussion injury (FPI)	ND				
Monkey/ macaque	Repeated mild	ND				
Monkey/ macaque	Penetrating ballistic-like brain injury	ND				
**Mice (** ***Mus musculus)***						
C57BL/6, CD1, BALB/c	Fluid percussion injury (FPI)	Focal or diffuse	Weight loss, seizures	Diminished cognitive and motor capabilities, including deficits, in spatial learning and memory (not shown in BALB/c mice), altered righting reflex time, impaired vestibulomotor and sensorimotor function, increased neurological severity score (NNS), decreased mechanical sensitivity, depression like behaviors	Contusion, hematomas, tissue lacerations, diffuse axonal injury, gliosis, edema, increased intracranial pressure, macrophage recruitment, hippocampal neurodegeneration, BBB disruption	Stelfa et al., 2022; Carbonell et al., 1998; Alder et al., 2011; Bolkvadze and Pitkanen, 2012
C57BL/6, CD1	Controlled Cortical Impact (CCI)	Focal	seizures	Diminished cognitive and motor capabilities, including deficits in spatial learning and memory	Neurodegeneration, reduced cortical volume, increase in mossy fiber density, BBB disruption, axonal injury, subdural hematoma, selective tissue loss in the hippocampus, astrocyte activation	Bolkvadze and Pitkanen, 2012; Smith et al., 1995; Xu et al., 2019
C57/BL6	Weight drop	Focal	Weight loss	Diminished cognitive and motor capabilities, including deficits in spatial learning	BBB disruption, amyloid B deposition, cerebral edema, neuronal death	Yang et al., 2013; Shishido et al., 2019
C57BL/6	Blast Injury	Diffuse	Lower nesting scores	Diminished cognitive and motor capabilities, including deficits in spatial learning and righting time. Increased anxiety-like behavior	BBB disruption, reactive gliosis, cellular edema, abnormalities in astrocytic end foot and tight junctional structure, elevated phosphorylated tau levels, myelin sheath deficits, axonal injury, mitochondrial abnormalities, altered hippocampal electrophysiology	Logsdon et al., 2018; Beamer et al., 2016; Li et al., 2023; Konan et al., 2019; Cernak et al., 2011; Song et al., 2018
**C57BL/6, B6C3F1**	Repeated mild	Diffuse	Weight loss	Diminished cognitive and motor capabilities, including deficits in spatial learning and memory, increased anxiety- like behavior and risk-taking behavior	Astrogliosis, microglial activation, white matter pathology, neuronal loss, mitochondrial impairment, axonal injury	Broussard et al., 2018; Gold et al., 2018; Robinson et al., 2017; DeFord et al., 2002; Hubbard et al., 2019
C57BL/6	Penetrating ballistic-like brain injury	Focal	Weight loss	Diminished motor capabilities	Neuronal degeneration, BBB disruption, immune cell infiltration, brain cavitation, astrogliosis, microglial activation	Cernak et al., 2014

ND, not described.

Animal models utilized for TBI research include rats, mice, pigs, rabbits, dogs, swine, sheep, ferrets, monkeys, and cats, which have been evaluated and critiqued by others (Xiong et al., 2013; Cernak, 2005; Ma et al., 2019). Non-human primate models of TBI are essential for accurate modeling of TBI impact on neural damage as these models most closely resemble the human brain; however, these models are limited (Barbay et al., 2021). The most common model of TBI is the rodent (mouse and rat), which we will focus on in this review. Additionally, it is essential to consider that TBI is a heterogeneous injury and that there are many injury models that replicate various pathologies, neurological manifestations, and severity levels. While the classification of TBI is under current refinement including the use of endophenotypes, we aim to review several known severity categories in humans (mild, moderate/severe) and compare human disease pathology to rodent models.

#### 2.2.1 Mild TBI

Mild TBI is considered a score of 13–15 on the GCS. Mild TBI is the most common type of TBI, with 70%−90% of treated brain injuries falling into the mild category (Cassidy et al., 2004). Furthermore, it is estimated that the incidence of treated mild TBI is 100–300/100,000, however, the true incidence is likely much higher since many mild TBIs are not treated at a hospital (Cassidy et al., 2004). Risk factors for mild TBI include intoxication, low education, age, intimate partner violence, military deployment, contact sports, and socioeconomic status (Alexis et al., 2022; Nordstrom et al., 2013; Gardner and Yaffe, 2015). Frequently, mild TBIs show no abnormalities on CT or MRI scans, yet many patients suffer from a plethora of symptoms. Common symptoms of a mild TBI include post traumatic amnesia, loss of mental alertness, anterograde amnesia, confusion, speech and gait abnormalities, personality changes, a lack of energy, and sometimes a loss of consciousness. Various rodent models of mild TBI, including controlled cortical impact (CCI), weight drop, closed head injury, and fluid percussion injury (FPI) models display many of the outcomes seen in humans (Table 3) (Bodnar et al., 2019). These symptoms included diminished cognitive and motor capabilities, including deficits in spatial learning and memory, increased anxiety-like behavior, and risk-taking behavior. Further, many of the pathological changes in human cases were replicated in mouse models. Pathological changes include accumulation of phosphorylated tau, white matter structure abnormalities, diffuse axonal injury, inflammation, and BBB disruption (Xu et al., 2021; Wu et al., 2020).

#### 2.2.2 Moderate and severe TBI

Moderate and severe TBIs are classified by having GCS scores of 9–12 and 3–8, respectively. Falls are a leading cause of moderate and severe TBI, particularly for older adults (Iaccarino et al., 2018). Motor vehicle accidents, assaults, and firearm-related injuries also account for many moderate and severe TBIs (Iaccarino et al., 2018; Miller et al., 2020). Loss of consciousness is typically longer with moderate and severe TBI and can last between 30 min and 6 h for moderate TBI and even over 6 h for severe TBI. Common symptomology includes general cognitive impairment, dizziness, confusion, seeing stars, no memory of the injury, persistent or worsening headache, vomiting, nausea, seizures, dilations of pupils, fluid draining from the nose or ears, and/or inability to wake from sleep. Pathological findings can include subarachnoid hemorrhage, subdural hematoma, extradural hematoma, intraventricular hemorrhage, effacement of ventricles, cerebral edema, bradycardia, elevated blood pressure and intracranial pressure, midline shift, cerebral contusion, skull fracture, cerebellar damage, BBB disruption, hippocampal volume loss, increased serum cytokine levels, elevated Glial fibrillary acidic protein (GFAP), myelin basic protein (*MBP*), and neurofilament light (NfL) deposition. Long-term neurological and psychiatric consequences can include general cognitive impairment, schizophrenia, depression, hallucinations, anxiety, substance abuse, somatoform disorder, adjustment disorder, affective disorder, and general psychiatric diagnosis. Numerous rodent models of moderate and severe TBI have recapitulated human TBI pathology and behavioral deficits (Table 3) (Yu et al., 2009; Pischiutta et al., 2018; Mao et al., 2020).

### 2.3 NA

Early descriptions of human exposure to NAs occurred through laboratory exposure through inhalation of tabun (ethyl N,N-dimethylphosphoramidocyanidate) (Lopez-Munoz et al., 2008). Exposure to sarin (isopropyl methylphosphonofluoridate), soman (pinacolyl methylphosphonofluoridate), and VX are the most well documented in both patients and animal models (Moshiri et al., 2012). Unlike the aforementioned viruses or TBI models, the mechanism of action and the manifestation of disease is nearly identical across different NAs. The primary mechanism of action of all NAs is the inhibition of acetylcholinesterase activity, leading to accumulation of acetylcholine at neuronal synapses. The excess acetylcholine, termed cholinergic crisis, causes prolonged activation of nicotinic and muscarinic receptor activity, which induces neurotoxic symptoms including muscle cramping, paralysis, headaches, and more (Lopez-Munoz et al., 2008). The accumulation of acetylcholine at the synapses in both the peripheral nervous system (PNS) and CNS leads to widespread CNS damage, with damage in the limbic system, particularly the amygdala and hippocampus, being most noted in the literature (Prager et al., 2014; Shih et al., 2003; Miller et al., 2015). The dentate gyrus of the hippocampus is built of tightly packed cholinergic neurons, therefore, they are highly sensitive to acetylcholine accumulation. Communication between the amygdala and the hippocampus is essential for memory and anxiety behaviors, and these cholinergic neurons play a large role in signal transmission between these portions of the brain (Song, 2023); therefore, it is unsurprising that these regions are significantly impacted by nerve agents (Aroniadou-Anderjaska et al., 2009). While the action of all NAs is similar, the primary differences in disease manifestation are related to the concentration and duration of exposure (CDC, 2017). Unfortunately, high or prolonged low-dosage exposure is highly fatal without immediate medical intervention to mediate seizure damage. The G-series agents (soman and sarin mostly discussed here) are water-like in consistency and easily form vapors, while the V-series agents (VX primarily discussed here) which are a thicker consistency and typically do not vaporize, leading to prolonged presence in the environment (Fan et al., 2024). While not discussed here, it's worth noting that several research articles have used surrogates, which are nonvolatile chemical compounds that inhibit acetylcholinesterase activity but are not as toxic or as strictly controlled as NAs (Finnegan et al., 2021). These compounds are valuable for enabling more laboratory research for therapeutics and biomarker analysis, but are not as toxic as G and V series nerve agents, so they are not the focus of this review.

In the following sections, we will review the animal models of NA exposure, which consist of NHP, rat, and mouse models. Animal models have been reviewed previously (Figueiredo et al., 2018; Pereira et al., 2014). As briefly discussed in future sections, an NHP model is ideal for nerve agent research because humans and primates have conserved nicotinic acetylcholine receptors (Kendrick et al., 2021). Cynomolgus, African green, and rhesus monkeys, as well as common marmosets have been investigated (Despain et al., 2007). Some models that have not been as well evaluated, such as the baboon model, have shown significant airway decline and neuromuscular junction activity, but the effects were inconsistent due to anesthetization with phenobarbital which inhibits some of the clinical presenting signs of NA toxicity, including muscle fasciculation and seizures (Anzueto et al., 1990). Collectively, it has been suggested that NHPs are the best model for NA intoxication due to similar levels of organophosphorus metabolizing carboxylesterases to humans though new humanized mouse models are being developed to better mimic human responses and reduce some of the ethical challenges of NHP experimentation (Marrero-Rosado et al., 2021; Tressler et al., 2024).

#### 2.3.1 G-series nerve agents (GA, GB, GD)

Several instances of G-series NA exposure have been documented in humans, but the extent of exposure and resulting neurological sequelae are not well characterized. Notable use occurred in Northern Iraq (Iraq, Iran War), Syria, and Damascus (Balali-Mood and Saber, 2012; Rosman et al., 2014; Thiermann et al., 1999). Documentation of sarin exposure in humans has been primarily on accidental exposures (Duffy et al., 1979) though several long-term studies of sarin exposure have been conducted on victims of the Tokyo and Matsumoto subway terrorist attacks (Duffy et al., 1979; Okumura et al., 1996). Inhalation and ingestion are the most toxic routes of NA exposure and induce faster, more severe symptoms than dermal contact which can take hours for symptoms to onset (Vucinic et al., 2017). Sarin, tabun, and soman are highly volatile and lethal doses are estimated to be between 10–500 mg-min/m^3^ (Table 4). Acute symptoms of exposure include headache, nausea, vomiting, and seizures (CDC, 2018). Prolonged symptoms of G-series exposure include depression, anxiety, mood swings, memory impairment, and cognitive and behavioral changes (Figueiredo et al., 2018; Levin and Rodnitzky, 1976; Rosenstock et al., 1991; Wesseling et al., 2002; Savage et al., 1988; Roldan-Tapia et al., 2005). Some evidence has suggested that Gulf War illness, characterized by broadly defined features such as tiredness, pain, memory impairment and imbalance in >250,000 warfighters, may be partially due to low levels of sarin exposure (Haley et al., 2022; Elhaj and Reynolds, 2023). These long-term effects are currently hypothesized to be due to intracellular cytotoxicity that correlates with the white matter edema observations in human cases following the Tokyo sarin attacks, but post mortem analysis of NA-exposed humans is limited (Bhagat et al., 2001, 2005). Collectively, early detection and antiseizure administration are the most crucial mediator of severe OP neurotoxicity (Gupta, 2020).

**Table 4:** Comparison of human cases and laboratory animal models of NA.

**Species/strain**	**Nerve agent**	**Dose (mg/kg animal models) (mg/m^3^/min humans)**	**50% lethal dose (mg/kg animal models) (mg/m^3^/min humans)**	**Acute symptoms**	**Neurological manifestation**	**Pathological changes**	**References**
**Humans**							
N/A	Soman	Unknown	50–500	Labored breathing, comatose/unconscious state, cyanosis, high blood pressure, increased heart rate, nausea, drooling, nasal drip, lock jaw, conjunctiva, muscle twitching or rigidity	Coma, hypochondriasis, hysteria, reduces motor control, reduced visual retention, photophobia, miosis	Not described	NRC, 1997; Sidell, 1974
N/A	Sarin	Unknown	2–102	Drooling, nasal dripping, cyanotic, convulsion, labored breathing, respiratory distress, wheezing, muscular fasciculations, decreased vision, nausea, vomiting, wheezing, cardiac abnormalities, tachycardia, voice loss, chest pains, heightened emotional state, abdominal pain, chest pain	Convulsion, headache, eye problems, sleep apnea, muscle soreness, behavioral and emotional changes (crying spells, depression, anxiety, fear), restlessness, fatigue, photophobia, coma, miosis, vegetative state	Cholinergic crisis	Duffy et al., 1979; Okumura et al., 1996; Nakajima et al., 1999; Abou-Donia et al., 2016; Okumura et al., 2005
N/A	VX	Unknown	10	Sluggish, malaise, muscle spasm, drooling, salivation, urination, vomiting, muscle twitch, cold sweat, flush face, nausea, vomiting, pallor	Drowsy, irritable, disorientation, delusions, hallucination, delayed speech, depression, fatigue, confusion, irritability, anxiety, insomnia, delirium, miosis	Not described	Bowers et al., 1964; Hayoun et al., 2024; Tu, 2020; Bramwell et al., 1963; National Research Council (US) Subcommittee on Chronic Reference Doses for Selected Chemical Warfare Agents, 1999
Macaque *(Macaca mulatta)*; Macaque *(Macaca fascicularis);* African Green Monkey (*Chlorocebus aethiops;* Baboons (*Papio cynecephalus anubis*)	Soman	1–8 LD_50_	5–15	Chewing, face automatisms, salivation, abstemious Increased salivation, chewing, muscle twitch, increased heart rate	Tremors, facial grimacing, seizure, convulsions, thrashing, sleep apnea	EEG abnormalities, cardiac arrhythmias, impaired hemodynamics, neutrophil influx, hypoxemia, decreased blood pressure	Despain et al., 2007; Anzueto et al., 1990; Raveh et al., 1997; Maxwell et al., 1992; Blick et al., 1991
Macaque *(Macaca fascicularis)*	VX	3 LD_50_	5–15	None observed/not reliable	None observed/Not reliable	None observed	Raveh et al., 1997; Lenz et al., 2005
Macaque *(Macaca fascicularis;* Marmoset *(Callithrix jacchus);* African Green Monkey (*Chlorocebus aethiops);* Baboons (*Papio cynecephalus anubis*)	Sarin	0.75–10 LD_50_	5–15	Difficulty breathing, nasal and oral secretion	Miosis, sleep apnea, convulsions, seizure	EEG abnormalities, cardiac arrhythmias, impaired hemodynamics, neutrophil influx, hypoxemia, decreased blood pressure	Anzueto et al., 1990; Woodard et al., 1994; Chapman et al., 2006; Abou-Donia et al., 2016; Van Helden, 2002; Genovese et al., 2007
**Rat** ***(Rattus norvegicus)***							
Sprague Dawley	Soman	1–1.5 LD_50_	50–200	Salivation, chewing, defecation, urination	Facial clonus, tremors, body jerks, straub tail fasciculation, Status epilepticus	Cell loss in hippocampus and piriform cortex, cellular remodeling throughout the brain, fiber degeneration in cortex, thalamus, amygdala, and fiber tracts, neurotoxic cytokine upregulation, upregulation of necrosis factors, microglia and astrocyte, activation, neuronal death	Bhagat et al., 2005; de Araujo Furtado et al., 2010; Prager et al., 2014; Myhrer et al., 2005; Kadar et al., 1995; Marrero-Rosado et al., 2018; Johnson and Kan, 2010; Shih et al., 1990; Johnson et al., 2015
Sprague Dawley	Sarin	0.5–1 LD_50_	100–180	Excessive salivation, lacrimation, urination, defecation	Tremor, respiratory distress, convulsions, seizure	Increased BBB permeability, neuron degradation in the cortex, hippocampus and cerebellum, increase in inflammatory markers, severe damage to the hippocampus, piriform cortex and thalamic nuclei	Kadar et al., 1995; Chapman et al., 2006; Abdel-Rahman et al., 2002
Sprague Dawley	VX	0.01–2 LD_50_	100–150	Often asymptomatic, weight loss, reduced grooming, diarrhea	Irritability, Aggression, seizure	Decreased hemoglobin, hematocrit, corpuscular volume, corpuscular hemoglobin, reduced red blood cell acetylcholinesterase	Shih et al., 2003; Goldman et al., 1988; Shi et al., 2023; Shih and McDonough, 2000; Stigler et al., 2022
**Mouse (** ***Mus musculus)***							
C57BL/6, CD-1, BALB/c	Sarin	0.4–0.5 LD_50_	100–180	Often asymptomatic, Weight loss, slumped posture, abnormal gait, eyelid closure, breathing abnormalities	Seizure, convulsions, miosis	Decrease dopamine turnover, astrocyte activation in the hippocampus, hippocampus degeneration, neuronal loss, acetylcholinesterase inhibition	Furman et al., 2014; Abou-Donia et al., 2016; Oswal et al., 2013
C57BL/6, CD-1, BALB/c, DBA, C3H, CF-1, ALAS, CFW	Soman	0.1–2 LD_50_	80–170	Slumped posture, abnormal gait	Seizure, tremors, status epilepticus, convulsions, forelimb clonus or tonus	Microglia activation in the thalamus, amygdala, hippocampus, and piriform cortex, neuronal death, B-cell activation, EEG abnormalities	Marrero-Rosado et al., 2021; Clement et al., 1981

ND, not described.

Soman exposure in NHPs has been well-established in several models (rhesus, cynomolgus, baboons, African green) to induce tremors, seizures, and muscle spasms (Despain et al., 2007; Raveh et al., 1997; Woodard et al., 1994; Maxwell et al., 1992). Immediate symptoms include chewing, increased salivation, and facial spasms with symptoms progressing to limb twitching, persistent tremors, convulsion, seizure, thrashing, and slumped posture (Despain et al., 2007). Without medical countermeasures to mediate seizure and acute toxicity, NA exposure is typically lethal. The lethal dose reported in African green monkeys was ~7.15 μg/kg, which is consistent with ranges from 5–15 μg/kg in rhesus, cynomolgus, and baboons (Table 4). Seizure activity is closely correlated with administered dosage of soman, where lower dosages result in less severe seizures and other neuromuscular changes, while higher dosage animals are prone to more frequent and longer lasting seizures, apnea, and cyanosis (Despain et al., 2007). Apnea, cardiac arrhythmia, and decreased blood pressure following soman exposure have been most well characterized in NHPs (Despain et al., 2007; Raveh et al., 1997; Woodard et al., 1994; Maxwell et al., 1992). The prolonged activation of acetylcholine receptors in humans and NHPs induces fatal neurotoxicity and, ultimately, death.

Symptoms of nerve agent poisoning in rodents resemble that of humans and NHPs with respiratory challenges, involuntary secretions, chewing, salivation, diarrhea, muscle fasciculation, tremors, convulsions, and seizures (Table 4). Soman can cross the BBB rapidly leading to increases in acetylcholine followed later by glutamate and subsequent neurotoxicity (Bhagat et al., 2005; Shih and McDonough, 1997). A single high dose of sarin or soman induces neuronal loss in rats dependent on seizure activity (McLeod et al., 1984; Petras, 1994). Pathological signs of injury occur soon after injury (4 h) but appear to worsen over time with notably increased severity at 3+ months post injury in the hippocampus, piriform cortex, and thalamus (Kadar et al., 1995). Single doses of sarin induce edema widespread \throughout the brain (Testylier et al., 1999). Extensive pathology of soman-exposed rats with memory deficits showed significant loss of neurons and interneurons and an increase in activated astrocytes and microglia in the hippocampus 90 days post-exposure (Reddy et al., 2020; Marrero-Rosado et al., 2018). BBB disruption and permeability significantly increase with exposure to NA across the brain; however, these impacts appear to be acute while the dysregulation of acetylcholine and muscarinic receptors appears to persist beyond 90 days post-injury. Persistent BBB disruption following NA exposure has not been well documented. Currently, BBB disruption appears to be at its height in the acute phase post-exposure and may be associated with convulsions and seizure typically seen acutely following exposure (Gupta, 2020). However, BBB restoration following injury in general can take time and depends on several factors, including severity of disruption and neuroinflammation. NA exposure is likely not an exception to this. In addition to pathological markers, there has been significant evaluation of neurotoxicity in rodent brains at various timepoints post exposure that display increased cytokine production, oxidative stress, and locomotor activity which becomes more apparent with increasing concentration of nerve agent (Henderson et al., 2002; Abu-Qare and Abou-Donia, 2002; Nieminen et al., 1990; Johnson and Kan, 2010). A review of animal models exposed to soman highlighted that in both rat, guinea pig, and nonhuman primate NA exposure models, only animals with seizures developed neuropathology and these seizures are the best variable for predicting neuron loss (Abdollahi and Karami-Mohajeri, 2012; Jett et al., 2020).

Unique challenges of NA exposure model development are the differing baseline levels of cholinesterase activity between inbred and outbred rodent strains. It has been relatively well established that C57BL/6 mice and C3H/He have less acetylcholinesterase activity in comparison to DBA/2 and BALB/c mice (Atalayer and Rowland, 2010). Genetic differences, including cholinesterase levels, appear to contribute to NA toxicity, especially regarding lethal dose and percent mortality (Matson et al., 2018; Furman et al., 2014). An additional challenge is the presence of serum carboxylesterase activity in rodent models of NAs. These compounds aid in degradation of NAs, thereby reducing the total NA concentration, but this activity appears to be absent in human cases. Differences in rodent response to NAs could also be sex dependent, as neurodegeneration and gliosis 4 months post exposure to soman is more significant in female animals than male animals (Gage et al., 2021).

#### 2.3.2 V-series nerve agents (VE, VG, VM, VR, VX)

The largest evidence of VX exposure consists of nearly 100 warfighters presenting ill-defined symptoms, including “altered awareness,” reduced intellectual ability, slowed movements, anxiety, and confusion (Bowers et al., 1964). V- series NAs induce a variety of symptoms including seizure, salivation, urination, vomiting, spasms, muscle twitching, and tremors (Hayoun et al., 2024). Incidence of seizure is lower in both humans and animals exposed to VX or VR compared to the G-series agents (Shih et al., 2003; Fawcett et al., 2009). Similar to the other OPs, chronic evaluation of nerve agents in humans is limited, but amnesia and behavioral changes may persist years after exposure (Nozaki et al., 1995). While human cell culture models of NAs are limited, a unique study performed a microarray analysis of human neurons and astrocytes exposed to VX and soman, respectively, and identified significant upregulation of apoptosis and inflammatory cascades independent of *in vivo* hallmarks of seizures and acetylcholine dysregulation (Hoard-Fruchey et al., 2020). Gene expression changes of astrocytes were not dependent on the agent used, but neurons appear to be significantly separated with both datasets showing significant upregulation of genes associated with the inflammatory response. It should be noted though that cell death was not induced by VX or soman in either cell population.

Multiple models for VX exposure exist including goats, guinea pigs, swine and mice. Observations in these animals are consistent with dose-dependent impacts of G-series agents, where facial twitches, chewing, tremors, seizures, but appear to be brief and all animals typically survive (Fawcett et al., 2009; Langston and Myers, 2016; O'Donnell et al., 2011). Ultimately, NHP and guinea pig models, previously reviewed (Pereira et al., 2014), appear to be the most reliable over rat and mouse models. Of the rodent models of VX exposure, there were minor changes in acute toxicity, histopathology, and weight, leading to authors conclusion of unreliable results with subcutaneous administration of sublethal doses (Goldman et al., 1988; Atchison et al., 2001). Minimal exploration of chronic aspects of VX exposure have been explored.

## 3 Comparative neuropathology

Indicated throughout this review, there are overarching similarities across the three neuropathologies in terms of symptoms and pathology. In humans, the symptomatic manifestations of disease often include seizure, confusion, behavioral or emotional changes. In following sections, we will compare disease progression of EEVs, TBI, and NA.

### 3.1 Overlapping clinical sequelae in humans

Disease manifestations of EEVs, TBI, and NA include overlapping symptoms and neuropathologies, which highlight the potential for both research gaps in neurological disease mechanisms as well as therapeutic potentials. These illnesses induce mild symptoms such as fever, confusion, headache, shock, neck pain, vomiting, malaise, and chills and can progress to more severe symptoms such as stupor, left-sided weakness, thalamic enhancement, dysarthria, convulsions, seizures, paralysis, intellectual disability, as well as cognitive, motor, and behavioral changes (Figure 1). EEV infection and TBI have a few shared neurological sequelae, including intellectual disability, memory loss, and depression, while convulsions are shared amongst EEV infection and NA exposure. Neurological sequelae shared by TBI and NA exposure include loss of consciousness, slurred speech, partial or complete vision loss, fatigue, insomnia, and drowsiness. All three pathologies can result in paralysis, coma, muscle twitch, photophobia, sleep disorders, and seizures (Figure 1). Although these conditions impact a varying number of individuals worldwide, there are significant case fatalities and progression to neurological sequelae warrantying further evaluation (Table 1).

### 3.2 General immune response to injury and agent exposure

**Figure 2:**
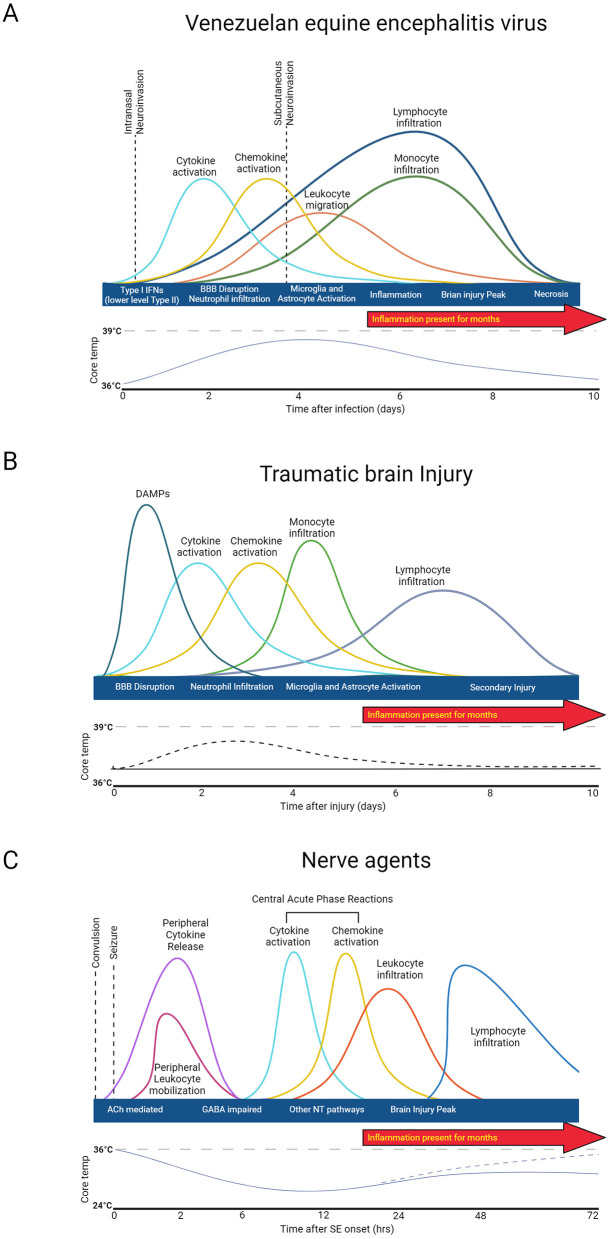
General immune response to injury and agent exposure. **(A)** Venezuelan equine encephalitis virus acute disease progression is biphasic. **(B)** Traumatic Brain injury damage consists of two phases: direct injury and secondary tissue damage. **(C)** Nerve agent damage is driven by neurotransmitter dysregulation. Created in BioRender. Kehn-hall (2024) BioRender.com/i57t162.

Immune system activation induced by NA, TBI, and EEVs follow relatively similar patterns; however, the timescale of activation based on the progression of the disease state varies drastically (Figure 2). In brief, primary acute damage driven by NAs occurs within the first 24 h and is primarily driven by immediate neurotransmitter dysregulation and seizures. Continued seizures after the initial injury can occur for days, months, or indefinitely if not mediated by antiseizure therapies. For TBI, the initial damage response recognition initiates a cytokine and chemokine response and glial cell activation in the first 4–5 days, but secondary damage can occur days and months after injury due to prolonged microglia activation and inflammation. EEVs initially evade the immune system and establish primary infection in lymphocytes in the first 4 days of infection, but as the virus spreads to the brain, there is an increase in immune mediators in both the PNS and CNS. While some is known about the immune response to NA and EEVs, much more is known about the immune response to TBI in both human and animal models.

While the immune response to EEVs has not been entirely elucidated, the replication and acute response to infection with VEEV have been explored (Skidmore and Bradfute, 2023). VEEV enters the cell via cell surface receptors, passes through the plasma membrane through endocytosis, and replicates within the host cytoplasm before genetic material is encapsulated and buds from the cell (Lundberg et al., 2017). All EEVs have a capsid protein which plays a significant role in dampening the host immune and antiviral responses to promote viral replication (Lundberg et al., 2017). Antivirals aimed at disrupting this activity of VEEV capsid have been tested *in vitro* (Thomas et al., 2018; Lundberg et al., 2017, 2018; Shechter et al., 2017; DeBono et al., 2019), but no data in animal models is available to date. Efficient replication strategies promote rapid spread into both PNS and established CNS infection, including in the brain within 24–72 h post-infection, depending on the route of infection (Phillips et al., 2016; Salimi et al., 2020) (Figure 2A). Both Type I and II interferon systems are likely initially upregulated by infection, but VEEV has been shown to disrupt nuclear localization of STAT1 and, therefore, reduce Type I and II Interferon systems. Treatment with IFNβ in both rats and NHPs significantly reduced VEEV levels in the CNS post-infection (Cwiklinska et al., 2020; Thorne et al., 2008).

VEEV infection in CH3 mice dampens cytokine levels at 1 DPI, whereas BALB/c mice have a slight upregulation at 1DPI and both models have significant upregulation of chemokines, cytokines, and upregulation of genes associated with interferon responsive genes by 6 DPI (Phelps et al., 2023). Five to six DPI, VEEV-infected mice also display decreased leukocytes and increased T-cells, monocytes, and neutrophils (Phelps et al., 2023). In addition to these gene expression studies, there have been some studies that have evaluated immune responses in mice in different rodent models and with a variety of different VEEV strains. It's proposed that attenuated strains of VEEV induce delayed cytokine expression profiles, but both attenuated and virulent strains of VEEV induce significant upregulation of INF-g, IL-6, IL-12, IL-10, and TNF-a (Grieder et al., 1997). IFNAR-1^−/−^ and IRF-2^−/−^ mice show accelerated viral replication, disease onset, and reactive oxygen species, highlighting that these genes play a role in viral dissemination and antiviral response (Schoneboom et al., 2000). Natural killer cells are early innate response immune effectors that induce IFN-y and cell death mechanisms (Taylor et al., 2012). Some reports suggest that natural killer cells also have neuroprotective roles; however, in VEEV, it appears that NK cells increase from 1 to 6 DPI and appear to worsen pathological outcomes of VEEV infection (Segal, 2007). Principal component analysis of a panel of chemokines, cytokines, and other immune and inflammatory markers distinctly separated VEEV-infected animals with pathology, highlighting that VEEV-induced pathology is highly correlated with inflammatory biomarkers (Phelps et al., 2023). Perivascular mononuclear cell infiltration, encephalitis, microglia activation, and neutrophil infiltration in the brain are well established at 7 DPI (Paessler et al., 2006). Collectively, it appears that CNS infiltration appears to occur at relatively the same time as T-cell, B-cell, Natural killer cells, neutrophil, and monocyte upregulation, only slightly later than cytokine and chemokine upregulation, which may promote severe CNS disease.

The time course of the immune response to TBI is biphasic (Figure 2B). Following the initial insult, tissue damage leads to the release of DAMPs (damage-associated molecular patterns). DAMPs stimulate resident cells to release chemokines and cytokines, which in turn recruit neutrophils to the injury site to contain the injury and remove debris. As the neutrophil population dwindles, monocytes begin to infiltrate, and glia become activated around the injury site to initiate reparative processes. T and B cells may also be recruited at later timepoints (Alam et al., 2020; Blennow et al., 2016; McKee and Lukens, 2016). Acute fever has been observed in one rodent models of TBI from day 1–4 post injury (Verduzco-Mendoza et al., 2023), which correlates with clinical case reports of fever after TBI, but this is inconsistently observed and not well characterized (Thompson et al., 2003). After injury, cytokines and chemokines are released, guiding circulating peripheral immune cells to the injury site. Microglia and astrocytes also become activated in response to injury and peripheral immune cell infiltration and can influence long-term outcomes (Loane and Kumar, 2016; Burda et al., 2016). This secondary injury can include but is not limited to, elevated intracranial pressure, ischemia, excitotoxicity, cell death, swelling, axonal injury, BBB disruption, and inflammation (Patel et al., 2023; Kochanek et al., 2000; Yi and Hazell, 2006).

Immune response after NA exposure is driven by convulsion and seizure damage (Figure 2C). Immediate consequences of nerve agent exposure are the inhibition of acetylcholinesterase, accumulation of acetylcholine at the synapse, and subsequent dysregulation of neurotransmitters GABA and glutamate function that leads to neurotoxicity (Figueiredo et al., 2018). Peripheral cytokine and leukocyte activation occur within 30 min post-seizure onset (de Araujo Furtado et al., 2012; Johnson et al., 2011). Proinflammatory cytokines (IL-1B, IL-6) and necrosis factors (TNF-α) increase as early as 2 h post-exposure to NA and peak between 6 and 24 h, depending on the agent and exposure concentration (Johnson and Kan, 2010; Chapman et al., 2006). Inflammation is accompanied by microglial and astrocyte activation, CNS inflammation, and peak injury within the first 48 h (Zimmer et al., 1997). Long-term functional and structural damage primarily dependent primarily depends on the duration and frequency of seizure post-exposure (Hrvat and Kovarik, 2020).

### 3.3 Acute vs. chronic neurological manifestations of rodent models

Documentation of chronic pathological changes is limited in humans and NHPs; therefore, to eliminate these gaps, we primarily focus on rodent models. While assessment of acute neuropathologies is crucial for animal model development and evaluation of biomarkers; chronic manifestations of disease are less well-understood.

**Figure 3:**
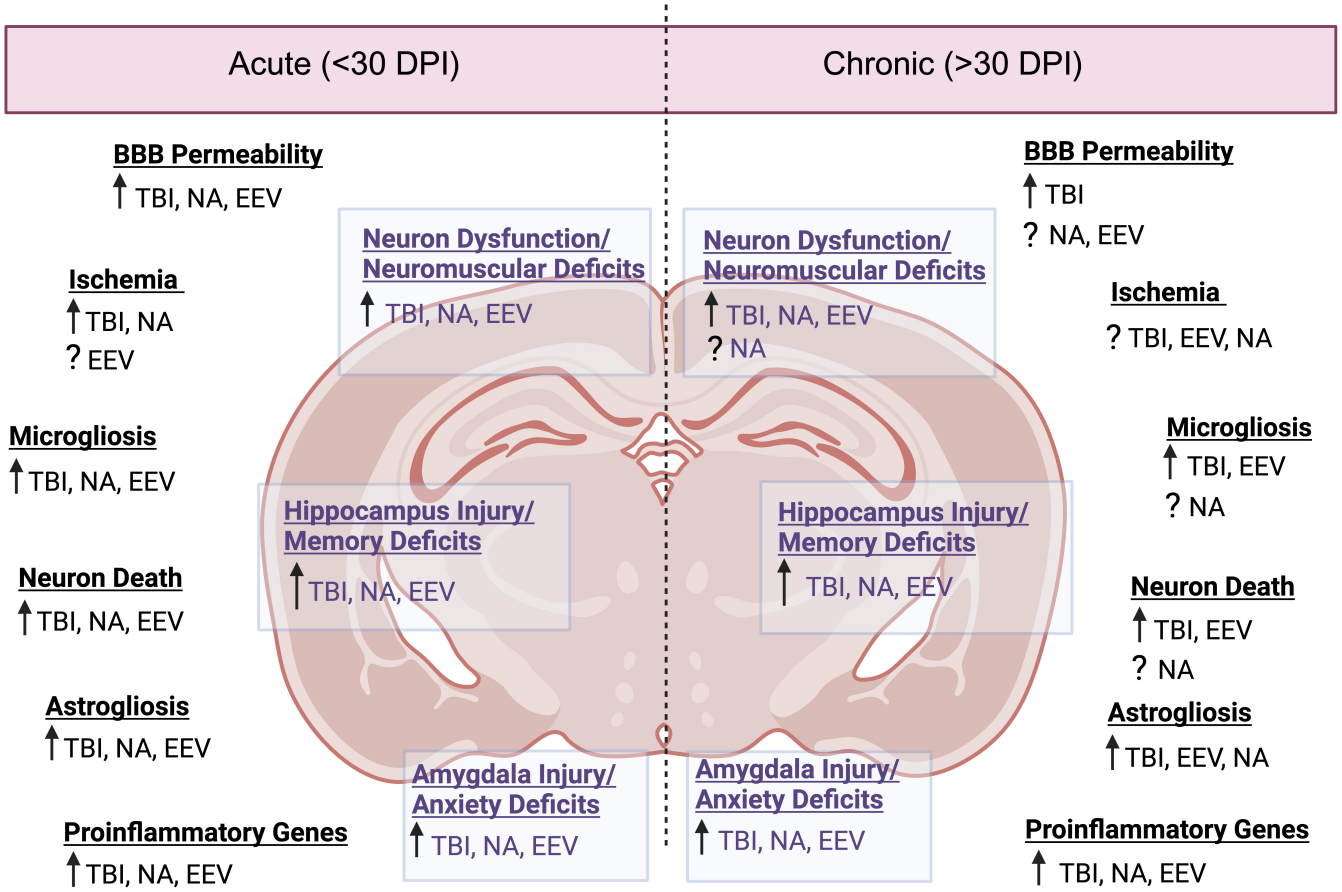
Neuropathology comparison between EEVs, TBI, and NA. Comparison of acute (< 30 days post injury or exposure) and chronic manifestations (>30 days post injury or exposure) of disease in rodent models. DPI, days post injury/infection, See Table 5 for associated literature for each observation. Created in BioRender. Kehn-hall (2024) BioRender.com/b05r121.

Throughout this review, it has been highlighted that EEV infection induces prolonged neurological symptoms. Acute infection of VEEV yields neuronal necrosis, lesions in the thalamus and olfactory cortex, and perivascular cuffing and gliosis with severe inflammation in the hippocampus and cortex (Reed et al., 2004; Gleiser et al., 1961; Danes et al., 1973; Victor et al., 1956) (Figure 3 and Table 5). While viral replication is not dependent on BBB breakdown, hemorrhage, lesions, and BBB breakdown are observed in acute infection, which could promote worsened chronic outcomes (Cain et al., 2017; Fongsaran et al., 2024). The overarching similarity between VEEV and EEEV infections in mice is the neuronal damage; however, following infection with VEEV, neurons in the hippocampus and cerebellum show signs of morphological changes correlated with apoptosis, whereas damage following EEEV infection seems to induce widespread necrosis, which is uniformly fatal (Steele and Twenhafel, 2010; Honnold et al., 2015a,b). Pathological evidence of vasculitis, perivascular cuffing, edema, hemorrhage, and widespread neuronal necrosis across the brain has also been observed in a variety of different models (Steele and Twenhafel, 2010). The chronic manifestations of EEVs have only recently been explored, but it is evident that VEEV induces neuronal loss, astrocyte activation, neuromuscular deficits, and fear responses several months post-exposure (Ronca et al., 2016, 2017; Fongsaran et al., 2024).

**Table 5:** Neuropathology comparison between acute and chronic manifestations of EEV, TBI, and NA in rodents.

	**Characteristic**	**VEEV**	**TBI**	**NA**
**Acute manifestations**				
Pathology changes	Astrogliosis	Yes (Ronca et al., 2017; Peng et al., 2013)	Yes (Cieri and Ramos, 2025)	Yes (Zimmer et al., 1997; Dhote et al., 2012; Baille-Le Crom et al., 1995; Filliat et al., 2007)
	Microgliosis	Yes (Hollidge et al., 2021)	Yes (Donat et al., 2017; Mouzon et al., 2012)	Yes (Zimmer et al., 1997; Dhote et al., 2012)
	Neuronal loss	Yes (Ronca et al., 2017)	Yes (Conti et al., 1998)	Yes (Aroniadou-Anderjaska et al., 2008)
	Blood brain barrier disruption	Yes (Cain et al., 2017)	Yes (Baskaya et al., 1997)	Yes (Ashani and Catravas, 1981; Carpentier et al., 1990; Sellstrom et al., 1985)
	Oxidative stress	Yes (Montiel et al., 2015)	Yes (Fesharaki-Zadeh, 2022)	Yes (Pazdernik et al., 2001)
	Ischemia	ND	Yes (Weil et al., 2021; Green et al., 2024)	Yes (McLeod, 1985)
	Hemorrhage/edema	Yes (Steele and Twenhafel, 2010)	Yes (Hellal et al., 2004)	Yes (Testylier et al., 1999; Pazdernik et al., 2001)
	Pro-inflammatory markers	Yes (Schoneboom et al., 2000; Peng et al., 2013)	Yes (Dalgard et al., 2012)	Yes (Johnson and Kan, 2010; Chapman et al., 2006; Svensson et al., 2001)
	Histopathological lesions	Yes (Salimi et al., 2020)	Yes (Kumar et al., 2013)	Yes (McCarren et al., 2020; Calsbeek et al., 2021)
Behavioral changes	Neuromuscular deficits	Yes (Ronca et al., 2017)	Yes (Mouzon et al., 2012)	Yes (Loomis and Johnson, 1966)
	Hyperactivity/aggression	Yes (Ronca et al., 2017)	Yes (Schwarzbold et al., 2010)	Yes (Allon et al., 2005)
	Anxiety/fear	Yes (Ronca et al., 2017)	Yes (Schwarzbold et al., 2010; Pandey et al., 2009)	Yes (Sirkka et al., 1990; Baille et al., 2001; Francois et al., 2022)
	Memory loss	Unknown	Yes (Mouzon et al., 2012)	Yes (Filliat et al., 1999)
**Chronic manifestations**				
Pathology changes	Astrogliosis	Yes (Ronca et al., 2017)	Yes (Furman et al., 2014; Cieri and Ramos, 2025; Rogers et al., 1997)	Yes (Filliat et al., 2007; Collombet et al., 2005)
	Microgliosis	Yes (Fongsaran et al., 2024)	Yes (Donat et al., 2017)	ND
	Neuronal loss	Yes (Ronca et al., 2017)	Yes (Conti et al., 1998; Rogers et al., 1997)	ND
	Blood brain barrier disruption	ND	Yes (Baskaya et al., 1997; Glushakova et al., 2014)	ND
	Oxidative stress	ND	Yes (Fesharaki-Zadeh, 2022)	Yes (Pearson and Patel, 2016; Pearson-Smith et al., 2017; Pearson-Smith and Patel, 2020)
	Ischemia	ND	ND	ND
	Hemorrhage/edema	ND	No (Agoston et al., 2019)	ND
	Pro-inflammatory markers	Yes (Fongsaran et al., 2024)	Yes (Zheng et al., 2022)	Yes (Pearson and Patel, 2016; Pearson-Smith and Patel, 2020)
	Histopathological lesions	Yes (Ronca et al., 2017)	Yes (Osier et al., 2015)	Yes (McLeod, 1985)
Behavioral changes	Neuromuscular deficits	Yes (Ronca et al., 2017)	Yes (Leconte et al., 2020)	ND
	Hyperactivity/aggression	Yes (Ronca et al., 2017)	Yes (Eyolfson et al., 2020)	Yes (Allon et al., 2005)
	Anxiety/fear	Yes (Fongsaran et al., 2024)	Yes (Leconte et al., 2020; Jones et al., 2008)	Yes (Francois et al., 2022; Choi et al., 2013)
	Memory loss	Yes (Fongsaran et al., 2024)	Yes (Dixon et al., 1999; Luo et al., 2014)	Yes (Kassa et al., 2001; Filliat et al., 2007)

ND, not determined.

Despite varying mortality rates and animal models associated with TBI injury (including ferret, mouse, rat, dog, sheep, swine, monkeys, and more) there are a variety of pathological changes which are consistent across the models, including cortical, hippocampal, thalamic degeneration, neuronal death, glial activation, neuroinflammation, excitotoxicity, and BBB disruption (Blennow et al., 2016; Loane and Faden, 2010; Liu et al., 2010) (Figure 3 and Table 5). Following the primary insult, ‘secondary injury' will begin to occur in the minutes, hours, days, weeks, and years post-injury. This secondary injury can include but is not limited to, elevated intracranial pressure, ischemia, excitotoxicity, cell death, swelling, axonal injury, BBB disruption, and inflammation (Burda et al., 2016; Loane and Faden, 2010; Dewan et al., 2018).

For NAs, the severity of the disease is almost exclusively dependent upon the duration of SE post-exposure (Aroniadou-Anderjaska et al., 2008). NA-induced pathology includes severe damage to the amygdala, the thalamus, and the hippocampus (Aroniadou-Anderjaska et al., 2008) (Figure 3 and Table 5). Neuronal loss, neuronal necrosis, and neuronal lesion abnormalities have also been observed (Redell et al., 2020; Apland et al., 2017). In models where prolonged seizures continue for longer durations without medical intervention, the degree of neurodegeneration-induced mortality is very high; therefore, the majority of studies provide a medical countermeasure against seizures to study sequelae.

Collectively, there are signs of neuroinflammation and neuronal loss across all of these models (Figure 3). Additional shared neuropathologies include gliosis, disruption of the BBB, and hippocampal damage. Perhaps the most striking and consistent deficits between the three conditions are associated with regions of the brain associated with memory, namely the hippocampus. This suggests that there are shared neurological pathways altered by infection or exposure, which medical countermeasures can target to alleviate long-term sequelae.

### 3.4 Behavioral alterations in response to injury and agent exposure

In human cases, symptoms of EEVs, TBI, and NAs often result in behavioral and emotional changes, but the mechanisms behind these personality alterations induced by injury are relatively unknown. Animal models are crucial for developing a better understanding behind the underlying cause and mechanisms which drive these responses (van der Staay et al., 2009). In animal models, neuron damage as a result of NAs, TBI, and EEVs is well documented across the brain with the most striking impacts in the hippocampus, amygdala and other regions of the limbic system (Williams et al., 2023; Shih et al., 2003; Aroniadou-Anderjaska et al., 2008; Palmer et al., 2016; Atkins, 2011). In recent years, there has been an increased interest in memory and anxiety behaviors in rodents and how this information can better inform therapeutic development, including related to how potential therapeutics impact areas of the brain that drive these interactions. In both rodent and human brains, memory formation occurs through three main steps: encoding, storage, and recall, which primarily occurs between sensory signal input from the cortex, signal transduction through the limbic system, and storage within the hippocampus (Joshi et al., 2019).

Behavioral tests to evaluate memory in rodents include both spatial and recognition memory tests, such as Novel Object Recognition, Morris water maze, y-maze and novel arm y-maze. In sarin-exposed (low dosage) and recovered rats with no visible weight difference or persisting symptoms, there is significant impairment of spatial learning and memory via the Morris water maze test (Shi et al., 2023). Further analysis of these animals showed significantly decreased acetylcholine activity in the mouse hippocampus and decreased dendritic spine density in the hippocampal neurons 21 days post-injury, supporting these behavioral deficits. Gene ontology analysis of differentially expressed genes highlighted significant upregulation of genes associated with neurodegenerative diseases, including Huntington's disease and Alzheimer's disease, and downregulation. On a more chronic timescale of 6 weeks post-injury, adult rats exposed to sarin showed poor spatial memory via Y-Maze and the Morris water maze in both animals presenting clinical signs and asymptomatic animals (Kassa and Vachek, 2002; Kassa et al., 2001; Filliat et al., 1999; Raveh et al., 2002). TBI memory deficits have been well established following mild and moderate TBI (Malkesman et al., 2013). The memory deficits appear to be primarily associated with challenges with working memory [reviewed in Paterno et al. (2017)] and more information could be gleaned through new technology such as opto- and chemo-genetics (Paterno et al., 2017). The primary behavior evaluated in most EEV models is neuromuscular function, not memory, as the biosafety challenges with setting up large maze systems in biocontainment adds several challenges and limitations. Several studies have highlighted there is significant viral invasion and damage to the hippocampus as early as 4 days post-infection (Ronca et al., 2017; Williams et al., 2023). Active avoidance, which evaluates fear memory, has been evaluated for VEEV-infected mice with mixed effects as they discovered a reduced latency between shock and escape compared to uninfected controls, but no differences between shock avoidance (Fongsaran et al., 2024). Other behavioral mazes, or the behavior response following EEV infection has not been explored.

Emotional instability and behavioral changes, such as anxiety, are a common complaint in inpatient cases of TBI, NA, and EEVs. In the brain, anxiety is most often associated with the limbic system, of particular importance is the amygdala, which receives information from the cortices and regulates fear, emotion, and motivation (AbuHasan et al., 2024). These changes have been most thoroughly explored in TBI rodent models. Anxiety behaviors of rodents following TBI have been previously reviewed (Malkesman et al., 2013; Tucker and McCabe, 2021). The majority of tests performed in TBI-inflicted animals report increased anxiety in both rats and mice, in both closed head, CCI, and FPI injuries, but there are some outliers (Tucker and McCabe, 2021; Tucker et al., 2017; Lapinlampi et al., 2020; Das et al., 2019). WEEV infection has often been likened to Parkinson's-like disease, and mice exposed to WEEV display abnormal movement gait and increased run duration, which could be indicative of both motor dysfunction and increased anxiety (Bantle et al., 2019). Unfortunately, there has been no specific investigation of anxiety in rodent models of EEVs, but fear memory tests indicate there is a potential alteration of fear anxiety pathways (Fongsaran et al., 2024). At both acute and chronic timepoints following sarin and soman exposure, surviving rats show increased anxiety-like behavior, assessed via open field and elevated T-maze, at both acute and chronic sarin and soman exposure (Sirkka et al., 1990; Baille et al., 2005; Mamczarz et al., 2010). Recordings of other rodent anxiety-like behaviors such as fleeing, hiding, ridged movement, or other prey responses to predators could provide further insight into anxiety in models where behavioral tests have not been performed (Zhao et al., 2023).

One important consideration for rodent research is the difference in behavioral outcomes in regard to the strain of animals being utilized. For example, sham-injured C57BL/6 mice scored significantly better than FVB/N and 129/SvEMS sham mice in motor function assessments (Fox et al., 1999). FVB/N and 129/SvEMS sham mice were not capable of learning in the Morris water maze or Barnes circular maze tasks. Further, C57BL/6 and BALB/c mice show distinct anxiety-like and depressive-like behaviors, pain perception, motor performance, and learning and memory (Mogil et al., 1999; Lucki et al., 2001; An et al., 2011; Garcia and Esquivel, 2018; Stelfa et al., 2022). These strain differences suggest one should carefully select rodent strains based on the research conducted. Collectively, there is indication based on neuropathology and reports of human clinical cases that there are changes in memory and anxiety which could be evaluated using rodent behavioral models.

## 4 Targeted therapeutics and neuroprotective countermeasures

### 4.1 Current treatments for EEVs

Currently, no publicly available therapeutics or antiviral treatments are licensed for use in humans infected with VEEV, EEEV, or WEEV. The WHO report indicates no ongoing clinical trials for vaccines or therapeutics against EEVs; however, a recent review of preclinical therapeutics for VEEV is available (Ogorek and Golden, 2023). Several groups have investigated direct-acting antivirals, vaccines, and supportive treatments for injury or inflammation resulting from EEVs (Thomas et al., 2018; Lundberg et al., 2017, 2018; Shechter et al., 2017; DeBono et al., 2019; Ogorek and Golden, 2023; Jonsson et al., 2019; Panny et al., 2023; Lehman et al., 2022; Saikh et al., 2020; Risner et al., 2019; Gall et al., 2018; Carey et al., 2018, 2020; Barrera et al., 2021; Bakovic et al., 2021; Ahmed et al., 2020). Given this, the current treatments are limited to supportive care, a costly treatment with up to $4.6 million-dollar life-time care reported by an EEV infected patient (Villari et al., 1995). In the United States, a live attenuated version of VEEV (VEEV TC83) has been previously used to vaccinate military personnel and at-risk laboratory workers; however, there has been an estimated < 30% of those vaccinated developing flu-like illness and adverse reactions (Tigertt et al., 1962; Sewell, 1995). Immunocompetent species of mice and NHPs display similar tropism and CNS infection with one of the hallmarks of infection and major contributing factors to mortality being neuronal damage. Monoclonal antibodies have been used to protect NHPs from VEEV-induced death if administered 48 h post-infection and show some promise as a potential treatment; however, there were mutations in the virus detected in NHPs treated 24 h post-infection (Burke et al., 2019). Thus far, most medical countermeasures of EEVs have focused on the inhibition of viral replication. Prominent antivirals include quinazolinone compound, CID15997213, which inhibits viral replication of VEEV and WEEV; and a ML336 derivate BGDR-4, which has demonstrated over 90% protection against VEEV and EEEV if administrated within 48 h post-infection; although there are potential concerns with viral resistance which is concentration dependent (Jonsson et al., 2019; Chung et al., 2014). CID15997213 targets the viral nonstructural protein 2 (nsP2) (Chung et al., 2014), which contains protease and helicase activities, and BDGR compounds target nsP4, the viral RNA dependent RNA polymerase (Skidmore et al., 2020). The most recent BDGR derivative developed by this group, BDGR-49, shows 70% and 100% protection from 10x LD50 of EEEV (FL93-939) and VEEV (TrD), respectively (Cao et al., 2023). While there are no direct neuroprotective drug treatments for VEEV, there are treatments that have been investigated to reduce neurological damage in viral infection and neurodegenerative diseases. These include pretreatment or co-treatment at infection with melatonin, which significantly reduces mortality, apoptosis, and oxidative stress (Boga et al., 2012; Montiel et al., 2015). Current treatments are challenged by the short window required for adequate inhibition of the virus as well as the potential for viral mutations to overcome antiviral mechanisms. Other challenges are due to the unique feature of illness where many of the human cases that document long-term neurological deficits have mild flu-like symptoms or no symptoms in the early viral infection period, making it challenging to diagnose and prevent. Given this, there is an urgent need for both characterization of CNS infection and neuroprotective treatment options for acute and chronic illness manifestations.

### 4.2 Current treatments for TBI

As of this publication, there are no FDA-approved medications for the treatment of TBI (Food and Drug Administration, 2021). However, numerous interventions are used in the clinic to alleviate pain and symptomology. Like many other injuries, the elevation of the head has immediate effects as intracranial pressure is reduced as the cerebral spinal fluid is displaced, and venous outflow is increased (Sattur et al., 2023). Intracranial pressure monitoring using an instrument inside the patient's body is an additional option for those who show substantial neurological compromise but do not require immediate surgical intervention (Shim et al., 2023). Hyperventilation is a strategy used to reduce cerebral blood flow via vasoconstriction (Gouvea Bogossian et al., 2020). This is typically only used in severe cases when acute neurological decline is expected to occur. Antiepileptics are often used during the acute phase. However, there is no evidence that these drugs help to prevent PTE in the long term (Chang et al., 2003). The last resort in managing the injury is putting the patient into a medically induced coma, which reduces the metabolic demand in the brain (Galgano et al., 2017). In the most severe cases, often due to cerebral edema and/or severe bleeding, surgical intervention may be warranted. Surgical intervention typically involves a craniotomy over the brain region of interest, followed by the removal of the hematoma and vessel cauterization (Galgano et al., 2017). Altogether, the lack of FDA-approved medication or therapeutics suggests a need to better understand the underlying pathology.

### 4.3 Current treatments for NAs

Positive outcomes of NA illness induced by acute exposure are heavily reliant upon quick identification of the exposure and immediate treatment. In cases of high-dosage exposure to NAs, or a delay in the time between exposure and treatment, current treatment methods are inadequate at reducing or preventing long-term neurological deficits. Emerging NA biomarkers are crucial for quick identification of NA exposure which could aid in faster and more target specific treatments (Wang et al., 2022). Current treatments for NA-exposed patients typically consist of anti-seizure agents (i.e. benzodiazepine anticonvulsants such as diazepam and midazolam), atropine (a muscarinic acetylcholine receptor antagonist), and pralidoxime (a.k.a 2-PAM, an acetylcholinesterase reactivator) (Figueiredo et al., 2018; Lallement et al., 2001). Benzodiazepines are well characterized in the treatment of NA-induced seizure and are therefore the anticonvulsant drug of choice, while neurosteroids are only recently being explored (Reddy, 2024). Neurosteroids may be an effective treatment for NA-induced seizure, especially seizure that has become refractory to benzodiazepine treatment. However, more research is needed. Combination treatments such as combining the NMDA antagonist ketamine with anticholinergic medications such as atropine have indicated improved outcomes compared to just atropine alone (Marrero-Rosado et al., 2021). Prophylactic treatments, such as carbamate pyridostigmine bromide, have been explored as protective agents against inhibited cholinesterases (Kassa et al., 2001; Bajgar, 2004; Kassa et al., 2008; Gordon et al., 1978). Emerging anticonvulsant options are also being explored, including the neurosteroid, Ganaxolone, which aids in reactivation of GABA-A receptors (Reddy, 2024). Neurosteroids are a promising alternative to benzodiazepines to mitigate acute and refractory seizures. Collectively, several potential therapeutics focused on acetylcholinesterase inhibition could be utilized to pretreat against nerve agent toxicity (van Helden et al., 2011); however, the approval of these therapies are still in the early stages.

### 4.4 Neuroprotective therapeutics

Neuroprotective therapies have been broadly defined as therapies that either reduce, prevent, or reverse permanent damage to neuron structure and/or function (Levi and Brimble, 2004; Mallah et al., 2020). Most commonly, neuroprotective agents mediate conditions such as Alzheimer's, Parkinson's, or Ischemia (Rehman et al., 2019). As discussed earlier, there are many therapies in the preclinical evaluation stage and some emerging therapies in later stages of the clinical approval pipeline which may be able to mediate some of the neurological deficits caused by EEV, TBI, and NA damage. Currently, the DrugBank online resource cites 43 drugs which are classified as neuroprotective agents (Knox et al., 2024; Wishart et al., 2018; Law et al., 2014; Knox et al., 2011; Wishart et al., 2006).

One area of potential therapeutic development would be guided toward novel treatments selective for modulating neurotransmitter activity to improve neuron signaling. Acetylcholinesterase therapies have proven beneficial in antipsychotic therapy of schizophrenia, as well as visual hallucinations and dementia in Parkinson's and Alzheimer's disease (Singh et al., 2012; Bittner et al., 2023). Gacyclidine, an NMDA receptor agonist, is a psychoactive drug that has been evaluated for a variety of neuroprotective mechanisms, including against OPs. One study evaluated GK-11 (Gacyclidine) treatment in NHPs exposed to OP levels at 8x the LD50 and found it protected animals from mortality (Golime et al., 2018). Similarly, there are a variety of neurotransmitter mediating therapies that aid with both negative regulation of signal transduction, such as Ziconotide which mediates chronic pain via calcium channel blockage (McGivern, 2007), and Tenocyclidin which mediates NMDA binding and provides anesthetic and dissociative effects in a variety of different situations including mediation of severe injuries such as spinal cord or brain injury (Radic et al., 2006). Mediation of the toxic effects of neurotransmitter dysregulation could help preserve neuron function and general immune responses to promote clearance of damaged regions of the brain following injury.

Oxidative stress is a driving mechanism behind many progressive neurodegenerative diseases as well as a potential therapeutic avenue against secondary injuries. Mitochondrial disruption is one of the drivers of this damage and has been identified in EEV (Keck et al., 2018), TBI (Hiebert et al., 2015), and NAs (Pearson and Patel, 2016). Antioxidants can help with scavenging and clearing radical oxygen species in the brain, as well as promoting the stability of naturally occurring antioxidants during phases of mitochondrial dysregulation (Ashok et al., 2022). Several antioxidants have been evaluated in clinical trials against neurological injuries and appear to be a safe and well-tolerated therapeutic option, these have been previously reviewed (Lee et al., 2020; Kelsey et al., 2010; Lalkovicova and Danielisova, 2016; Teleanu et al., 2019).

Combating the death of healthy cells while clearing damaged or misfunctioning cells is a major challenge for every organism. Neurodegenerative diseases almost always have alterations in programmed cell death (apoptosis), mitochondrial recycling (mitophagy), or cellular recycling (autophagy) mechanisms (Knox et al., 2024). For VEEV it has been shown there is an upregulation in apoptosis and mitophagy which ultimately worsens pathology in response to infection (Baer et al., 2016; Keck et al., 2017). Several cell death pathways are induced by TBI and OPNA and are likely a leading cause of secondary injury (Wu and Lipinski, 2019). While these mechanisms require energetic stress and cause the death of cells, autophagic mechanisms are often beneficial as they promote restoration of cellular function (Liao et al., 2022). Therefore, modulating cell death or recycling mechanisms is a challenge, and short-term administration of potential therapeutics could be an avenue of healthy cell death while also promoting the uncontrolled growth of dysfunctional cells. In both spinal cord injury and TBI, rapamycin treatment reduces neuron death through activation of autophagy and microglia activation (Husain and Byrareddy, 2020; Song et al., 2015; Li et al., 2019). In combination with lithium, rapamycin can provide synergistic effects inducing autophagy to enhance the clearing of protein aggregates associated with aging neurons (Sarkar et al., 2008; Cherra, 2008). Apoptosis, driven by caspase-3 activation, is associated with apoptosis in the CNS as well as several diseases, including TBI, spinal cord injury, and Alzheimer's disease (Khan et al., 2015). Activation of the neuronal-specific inhibitor of the apoptosis (IAP) family has been shown to reduce the loss of hippocampal neurons, which could potentially reduce pathology and memory deficits (Robertson et al., 2000). Further evaluation of apoptosis and autophagy mechanisms is necessary to determine specific time points post-injury where these therapies could be beneficial or harmful to patient outcomes.

While this is not an exhaustive list, these neuroprotective therapies highlight crucial areas for advancement in neuroprotective health. Further studies into the mechanisms behind neurological damage, especially those that target deficits common across multiple neurodegenerative diseases, such as memory loss or anxiety, could provide promising avenues for therapeutics.

## 5 Comparative neurodegenerative diseases

### 5.1 Alzheimer's disease

Alzheimer's disease is the most common neurodegenerative disease driven by the accumulation of misfolded proteins. Unlike many other neurodegenerative diseases, the onset of Alzheimer's disease is not typically associated with genetic mutations but rather a combination of environmental and lifestyle (i.e., age and fitness) risk factors (Korczyn and Grinberg, 2024). Typically associated with increasing age, the initial symptoms of Alzheimer's disease are mild memory loss, which typically progresses into more severe memory loss over time, but each individual case has a different rate of disease progression. Therapeutic options for Alzheimer's disease are limited, but therapies currently include antipsychotics and mediators of abnormal neurotransmitter function which contribute to memory loss-induced psychosis (Eassa et al., 2023; Drevets and Rubin, 1989; Wang et al., 2023; Ricci et al., 2021). In recent years, there has been an increasing interest in the relationship between infectious agents and neurodegenerative disease due to a variety of shared factors, including neuron loss, increased cellular damage, and increased inflammation (Piekut et al., 2022). Memory deficits have been noted in clinical cases of NA, TBI, and EEV survivors. A recent study highlighted the similarities between VEEV and Alzheimer's utilizing early onset Alzheimer's animal model (Tg2576) revealed that alphavirus infection can speed up neurodegenerative phenotypes associated with Alzheimer's, including pro-damage cytokines TNF-alpha and IL-1B, fear memory formation, and amyloid beta plaque concentrations (Fongsaran et al., 2024). Similar links have been made between Alzheimer's disease and TBI, as there are signs of amyloid beta accumulation and an increase in the same cytokine profiles; however, it is still unclear what leads to the accumulation of these factors beyond the hypothesis that its driven by vascular dysfunction or ischemic damage from BBB disruption (Ramos-Cejudo et al., 2018). Several reversible acetylcholine esterase inhibitors have been utilized to treat Alzheimer's disease and have been proposed as potential prophylactic therapeutic options for acetylcholinesterase toxicity caused by NA exposure given that they can occupy acetylcholinesterase binding sites, therefore minimizing the effects of NA. One of these therapies, Galantamine, was used to rescue animals from death and provided notable prevention from sarin and soman-induced neurodegeneration (Golime et al., 2018; Albuquerque et al., 2006). Acetylcholinesterase-inhibiting therapies have greatly increased as a result of NA release, which can be used for the management of oxidative stress and neurotransmitter dysregulation caused by Alzheimer's disease through increasing acetylcholinesterase levels in the brain, though to a lesser extent than NAs (Singh et al., 2012). Collectively, the overlapping neuropathologies between Alzheimer's disease and the biological, physical, and chemical injuries discussed here further highlight the need for broad neuroprotective and neuroreparative therapies.

### 5.2 Parkinson's disease

Parkinson's disease is the second most common neurodegenerative disease and is typically characterized by the reduction of dopaminergic neurons partially attributed to mitochondrial alteration, inflammation, and alpha-synuclein aggregation (Pardo-Moreno et al., 2023). Since its earliest discovery, WEEV infections in humans have been often likened to Parkinson's disease and there is significant dopaminergic neuron loss and a-synuclein protein aggregation (Bantle et al., 2019, 2021). Increased rigidity, tremors, and slowed movement have been observed in patients, and further pathological evaluation identified chronic microglia and astrocyte activation as well as aggregation of alpha-synuclein and neuron death (Bantle et al., 2019, 2021). In WEEV-infected patients, a Parkinson's therapy which inhibits decarboxylases (levodopa and trihexyphenidyl) is effective at reducing clinical disease indicating these drugs may be able to aid with long-term deficits (Oertel and Schulz, 2016). Other Parkinson's related therapies, including Caramiphen, Bupropion, and Scopolamine, have been explored for protection against soman in rats because of their ability to antagonize cholinergic and glutaminergic fluxes (Myhrer et al., 2008, 2013). Ultimately, these therapies reduced cognitive impairment induced by soman seizures, but co-administration of these drugs is typically required prophylactically to see robust results.

### 5.3 Epilepsy and recurrent seizures

Recurrent seizures and epilepsy impact over 65 million people per year and have a variety of causative conditions (Fordington and Manford, 2020; Kanner and Bicchi, 2022). Benzodiazepines for antiseizure effects are most effective against NA-induced neurological deficits because, as discussed previously, seizures are the main cause of neurological damage. Emerging reversable cholinesterase inhibitor therapies which have been used for Parkinson's therapeutic such as physostigmine, are being evaluated for prophylactic effects against NA-induced damage with some success (Myhrer et al., 2008, 2013). Post-exposure therapies for NA revolve around the immediate administration of antiseizure medications as the standard of care, but these same antiseizure measures have only been minorly explored in other neuropathologies, including TBI and EEV infection. In the clinic, there has been the utilization of anticonvulsant therapy to treat reoccurring seizures post symptom onset from humans infected with EEEV, which promoted survival in three patients, but neurological deficits, including hemiparesis and psychomotor deficits, persisted (Carrera I. et al., 2013). In TBI, prophylactic administration of antiepileptic drugs is effective in controlling seizures post-moderate TBI if provided within 7 days post-injury; however, these therapies are not effective if administered after 7 days post-injury (Chang et al., 2003). The effect of antiepileptic therapies beyond the early acute phase of injury or infection appears to be poorly understood. Collectively, antiseizure therapies appear to be effective if administered prior to, or shortly after TBI, NA, or EEV, but are not useful against chronic symptoms. Although antiepileptic therapies have significantly improved over the last 150 years, these therapies still have reduced effectiveness overtime, which appears to also overlap with treating other recurrent seizure conditions; there are still major challenges with the development of tolerance over time, requiring increased dosage and increased adverse effects (Schmidt, 2009). Given these challenges, the development of robust and target selective anticonvulsive therapies are crucial for a large number of seizure-causing diseases.

### 5.4 Mood, sleep, and behavioral disorders

Mood disorders are one of the most common disabilities worldwide, of which depression and sleep disorders are 18% (Dulawa and Janowsky, 2019) and 20% of the population, respectively (Chattu et al., 2018). Insomnia, nervousness, depression, anxiety, and irritability are common among many neurological diseases including many of the ones listed previously, and EEV, TBI, and NA. Depressive disorders has become known as the most common comorbidity, accompanying neurological disorders as well as cardiovascular, inflammatory, and metabolic disorders (Kochanek et al., 2020). Few mechanisms are known for the major drivers of these behaviors, but there are several categories of depression with different causes, whether that be prior injury, genetic susceptibility, or environmental changes (Remes et al., 2021). Given this, it is not surprising that there are many anti-depressant therapies available, with 5 major types based on mechanisms of action. The most common typically mediate neurotransmitters are dopamine, norepinephrine, and serotonin. While there is much variability in the effectiveness of antidepressants and insomnia treatment, there are some areas of promise for elevating long-term symptoms caused by neurological disease. Melatonin has often been evaluated as a potential neuroprotective therapy to mediate oxidative stress and enhance sleep (Chitimus et al., 2020). Treatment with melatonin significantly reduces symptomatic disease, replicating virus in the brain, and mortality in mice infected with VEEV (Boga et al., 2012; Montiel et al., 2015; Ahmad et al., 2023). In TBI studies, melatonin has both anti-inflammatory and antioxidant properties which reduce secondary injury and chronic neurological deficits (Blum et al., 2021). Albeit a different mechanism of action, the steroid hormone 17β-estradiol, E2, has demonstrated neuroprotective qualities, including reducing TBI-altered miRNAs and reducing inflammation and depressive-like behavior in mice (Sell et al., 2019). Although not tested in NAs, melatonin and other steroid treatments can also be effective at reducing apoptosis, reducing inflammation, and controlling reactive oxygen which are protective in peripheral nerve injuries (Uyanikgil et al., 2017). Uses of melatonin as a treatment option for VEEV, TBI, and NA are currently limited to animal models, but the usage of this naturally occurring and affordable therapy in humans could be a valuable asset to mediating chronic damage induced by these conditions.

## 6 Conclusions and limitations

As discussed, there are a variety of animal models utilized to assess neurological changes induced by EEVs, TBI and NAs. One confounding variable presented throughout this review is the variation in animal models used between the models due to considerable cost differences and biocontainment challenges. In NHPs, symptoms including seizures, depression, and weight loss have been observed across TBI, NA, and EEVs, but pathology is limited. We can glean the most information from NHP models as these are the most similar to clinical outcomes in humans; however, the majority of the known information about these conditions comes from rodent models of infection.

Much of the research for EEVs has been performed *in vitro* in human or mouse cell lines and *in vivo* mouse models, whereas TBI and NA have in-depth *in vivo* rodent models which are more physiologically similar to humans and show more consistent and reliable results in behavioral tests. In rodent models, symptoms include weight loss, fatigue, and seizures. Pathological changes in the brain, including neuronal necrosis, astrocyte activation, inflammation, and blood-brain barrier disruption, are also common among these conditions in many areas, including those that mediate memory and anxiety.

While NHP models appear to be most similar to the human course of disease in TBI, NA, and EEVs, there is much more known about the transcriptomic and pathology changes in lower-order models such as mice and rats due to obvious cost and accessibility limitations. Some of the differences observed in NHP models compared to rodent models may be due to differing levels of neurotransmitters that mediate neuron communication and response to stimuli. NHPs have levels similar to those of the neurotransmitter acetylcholine in humans, while rodents typically have higher levels. This is attributed to worsened outcomes in rodent models exposed to NAs; however, it is unknown how levels of acetylcholine contribute to disease outcomes in EEVs.

Sex differences in humans and animal models is another important consideration in the TBI field. In the general population, data shows that men are more likely to sustain TBIs than women. Women report worse and more symptoms post injury, but research involving sex differences is impacted by fewer women than men being recruited or involved in work related injury (i.e. military services, motor vehicle injuries, competitive sports) (Gupte et al., 2019; Munivenkatappa et al., 2016). Within collegiate sports, women sustain more concussions than men who are engaging in the same sport. Further, there has been an increase in the number of women joining active military duty. Hereby, there is an increased risk of sport and combat related TBIs in women (Amoroso and Iverson, 2017; Covassin et al., 2016). The role of sex as a variable has been minorly explored in NA and EEVs, therefore; its beneficial to consider other neurological diseases for insight into how sex may play a role in disease outcome. The current literature suggests females may experience worsened disease in NA exposure (Sekijima et al., 1997). In VEEV exposure, more males have reported infection and a slightly higher percentage of neurological symptoms such as headaches, but it's this difference is not understood nor explored in animal models (Carrera et al., 2018). As discussed, sex as a variable has been briefly explored for TBI where women report worse and more symptoms post-injury, but this is impacted by fewer women than men being recruited or involved in work-related injury (i.e. military services, motor vehicle injuries, competitive sports) (Gupte et al., 2019; Munivenkatappa et al., 2016).

Age is also a confounding variable across the models. It has been well established that children infected with EEVs have significantly higher mortality and chronic behavioral changes compared to adults (Farber et al., 1940). In NA, younger exposed individuals can have more increased mortality and more severe responses due to different physiological changes in cholinergic stress, increased risk of respiratory distress, and reduced effectiveness of atropine requiring higher doses (Figueiredo et al., 2018; Klass and Westmoreland, 1985; Rotenberg and Newmark, 2003). Pediatric cases of TBI are devastating with higher levels of mortality and increased antisocial, depressive, and maladaptive behaviors beyond acute injury (Levin and Hanten, 2005; Andrews et al., 1998). Both age and sex are important variables for consideration of response to injury and therapeutics as there are notable differences in neuroanatomy and responses to pharmaceutical agents (Soldin and Mattison, 2009; McCarthy, 2016).

## 7 Future perspectives

Recent advances in our understanding of overlapping neurological pathways that drive chronic sequelae, disease outcomes, and associated behavioral changes have highlighted potential routes for interventional neuroprotective therapeutics. Despite having diverse causes, the biological, physical, and chemical injuries described have many similarities to other neurodegenerative diseases. More studies have been conducted on the chronic consequences of TBI due to vast number of individuals impacted by TBI expanding across military and civilian personnel. However, there have been limited studies to characterize the chronic consequences of EEVs and NAs in animal models. This is an area of great need to allow comparison between neurological sequelae observed in humans and in animal models. Recapitulation of neurological sequelae in animal models is critical to enable testing of therapeutics that can prevent neurological sequelae.

Expansion to include comparisons between all three disease/injury states should be considered given the potential of chemical and biological warfare threats. Cross-condition therapeutics which target secondary injury including inflammation, neuron loss, and BBB disruption could be targeted with therapeutics in acute phases of injury to promote survival and reduce long-term neurological deficits. Prophylactic measures could also be highly beneficial for military personnel going into areas of high-risk for physical, biological, or chemical agent exposure. Additional work evaluating the similarities of these therapies could reveal potential therapeutic countermeasures. Albeit not discussed in depth here, future studies which elaborate on integrative omics such as transcriptomic, epigenetic, metabolomic, and proteomic changes could aid in providing a better understanding of why neurological sequelae vary across individuals. Furthermore, there are several therapeutics mentioned in the literature that have promising neuroprotective qualities in rodent models inflicted with one type of injury or infection. Expanding these therapeutics to higher-order animal models or expansion of these therapeutics to other viruses, organophosphates, or physical injury could improve the drug discovery landscape and promote movement through the drug approval process more quickly.
